# Iron-Fur complex suppresses the expression of components of the cyclo-(Phe-Pro)-signaling regulatory pathway in *Vibrio vulnificus*

**DOI:** 10.3389/fmicb.2023.1273095

**Published:** 2023-10-03

**Authors:** Keun-Woo Lee, Soyee Kim, Sora Lee, Minjeong Kim, Suji Song, Kun-Soo Kim

**Affiliations:** Department of Life Sciences, Sogang University, Seoul, Republic of Korea

**Keywords:** *Vibrio vulnificus*, cyclo-(L-Phe-L-Pro), quorum-sensing, iron, Fur, virulence factors

## Abstract

In the human pathogen *Vibrio vulnificus*, the quorum-sensing (QS) signal molecule cyclo-(L-phenylalanine-L-proline) (cFP) plays a critical role in triggering a signaling pathway involving the components LeuO-vHUαβ-RpoS-KatG via the membrane signal receptor ToxR. In this study, we investigated the impact of iron on the expression of these signaling components. We found that the transcription of the membrane sensor protein ToxR was not significantly affected by Fur-iron. However, Fur-iron repressed the transcription of genes encoding all the downstream cytoplasmic components in this pathway by binding to the upstream regions of these genes. Consequently, the expression of genes regulated by the alternative sigma factor RpoS, as well as the resistance to hydrogen peroxide conferred by KatG, were repressed. Additionally, we observed that in *Vibrio cholerae*, genes dependent on ToxR showed higher expression levels in a *fur*-deletion mutant compared to the wild type. These findings indicate that iron, in association with Fur, represses virtually all the cytoplasmic components responsible for the ToxR-dependent cFP-signaling pathways in these two pathogenic *Vibrio* species. This study, along with our previous reports demonstrating the repression of components involved in AI-2 dependent QS signaling by Fur-iron, highlights the crucial role of iron in quorum-sensing regulation, which is closely associated with the pathogenicity of this human pathogen.

## Introduction

The opportunistic human pathogen *Vibrio vulnificus* is a halophilic Gram-negative bacterium that and causes primary sepsis in a certain high-risk human population (Kumamoto and Vukich, 1998). Like many other pathogenic bacteria, *V. vulnificus* employs complex signal transduction systems to sense various environmental factors and adjust corresponding functions accordingly for survival and pathogenicity (Kim et al., 2013b). A typical example is the quorum-sensing (QS) regulation, which is triggered by diffusible signal molecules produced by cognate bacterial cells and plays a significant role in regulation of sets of virulence factors.

A QS signaling pathway initiated by a diketopiperazine (DKP) signal molecule cyclo-(L-phenylalanine-L-proline) (cFP) has been identified in *Vibrio* spp., including pathogens *V. vulnificus, V. cholerae* and *Vibrio parahaemolyticus* as well as a non-pathogen *Vibrio harveyi* (Park et al., 2006; Bina and Bina, 2010). This signal is released from cells into environment through simple diffusion across the bacterial membrane (Park et al., 2020a) and the cFP signal is recognized in neighboring cells by the inner membrane receptor protein ToxR (Park et al., 2006; Bina et al., 2013), which is known to form either homodimers or heterodimers with ToxS (DiRita and Mekalanos, 1991). The transduction of the cFP signal from ToxR leads to induction of the expression of LeuO, the master regulator in the cFP-dependent signal pathway (Bina et al., 2013; Park et al., 2019). However, when the signal is not transduced, H-NS, a histone-like nucleoid structural protein, plays as a basal stopper role to suppress the expression of LeuO (Ghosh et al., 2006; Picker and Wing, 2016; Park et al., 2020b). LeuO, when over-expressed, exhibits a feedback control mechanism by inhibiting its own transcription (Park et al., 2019).

The LeuO master regulator subsequently activates the expression of several genes. This includes the porin OmpU (Park et al., 2006), as well as the histone-like proteins vHUα and vHUβ which enhance the post-transcriptional stability of the *rpoS* mRNA. The *rpoS* gene encodes an alternate sigma factor (Kim et al., 2018; Park et al., 2020b) that directs the transcription of a series of genes including *katG* encoding a catalase that confers resistance to hydrogen peroxide in the pathogen (Kim et al., 2018). Furthermore, transcriptomic analysis has revealed that more than 950 genes in *V. vulnificus* are modulated by cFP (Kim et al., 2013a), indicating that the cognate signal transductions exert a profound influence on physiology of the pathogen.

Iron is essential element for most living organisms including animals, plants, and bacteria (Schaible and Kaufmann, 2004; Waldron and Robinson, 2009). However, high concentrations of ferrous ion (Fe^2+^) can be toxic as they can generate highly reactive radicals through the Fenton reaction (Massé and Arguin, 2005). To survive, cells have developed strategies to maintain iron homeostasis in the cytoplasm. One common strategy employed by bacteria is the production of low-molecular weight compounds called siderophores, which have a high affinity for ferric iron (Fe^3+^) (Troxell and Hassan, 2013). In most bacteria, iron homeostasis is mainly regulated by the ferric uptake regulator (Fur) (Deng et al., 2015). Fur is crucial in host–parasite interactions as it controls the expression of various proteins involved in iron removal and uptake systems, which allow bacteria to acquire iron from heme or specifically internalize host iron-binding proteins (Vasil and Ochsner, 1999). *V. vulnificus* possesses a 143-amino acid Fur protein, which shares 79% homology with *Escherichia coli* Fur and 93% homologous to *V. cholerae* Fur (Litwin and Calderwood, 1993). Under iron-rich conditions, Fur-iron complexes typically recognize a DNA sequence called Fur box (5′-GATAATGATAATCATTATC-3′) which is present in the promoter region of target genes and affect RNA polymerase binding to modulate the expression of the genes (Wen et al., 2016).

We have demonstrated that the Fur-iron complex regulates the autoinducer-2 (AI-2) signaling QS pathway in *V. vulnificus*. The QS, along with Fur-iron complex, controls the production vulnibactin encoded by *vvsAB*, thereby maintaining the intracellular iron concentration at an appropriate level. Under conditions of iron limitation, the transcription level of *vvsAB* is low at low cell density but induced at high cell density. However, in the presence of iron, the Fur-iron complex represses the transcription of the genes regardless of cell density (Wen et al., 2012). We also have found that the Fur-iron complex also regulates virulence factors by modulate the expression of *smcR*, which encodes the master regulator of the AI-2 QS signaling system. This repression is achieved through the direct binding of the Fur-iron complex to the *cis*-acting element in the upstream region of *smcR* (Kim et al., 2013b). Five small RNA molecules called Qrrs1-5, which are involved in the QS regulation, are also regulated by the Fur-iron complex (Wen et al., 2016). We also have shown that the small RNA RyhB, which enhances the stability and translation of the LuxS mRNA responsible for produces AI-2, is inhibited by the Fur-iron complex (Lee et al., 2022).

Our findings suggest that iron antagonizes QS signaling, and the regulation is primarily mediated by the Fur protein. On the basis, we further investigated the impact of the Fur-iron complex on another QS system mediated by cFP signaling in *V. vulnificus*. In this study, we demonstrate that the Fur-iron complex exerts control over this signaling system as well, by repressing the expression of virtually all known components associated with the cFP signaling pathway. The results presented here underscore the significance of iron in the signaling mechanisms of *V. vulnificus* and, consequently, in the pathogenicity of this virulent species.

## Materials and methods

### Strains, plasmids, and culture conditions

The bacterial strains and plasmids used in this study are listed in Supplementary Table S1. *Escherichia coli* and *V. cholerae* strains were cultured at 37°C in Luria-Bertani (LB) broth supplemented with appropriate antibiotics. *V. vulnificus* strains were cultured in LB broth or thiosulfate citrate bile salt sucrose (TCBS) agar at 30°C. When necessary, either ferrous sulfate (25 μM) as an iron source or 2,2′-dipyridyl (100 μM) as an iron chelator was added exogenously to the LB broth when the A_600_ value of the culture reached approximately 0.1. Antibiotics were used at the following concentration: For *E. coli*, ampicillin 50 μg/mL, kanamycin 25 μg/mL, tetracycline 10 μg/mL, chloramphenicol 25 μg/mL; for *V. vulnificus* and *V. cholerae*, kanamycin 100 μg/mL, tetracycline 2 μg/mL, chloramphenicol 2 μg/mL. All media used in this study were purchased from Difco (MI, United States). All reagents and antibiotics were purchased from Sigma Aldrich (MO, United States).

### Construction of a *fur* deletion in wild-type MO6-24/O

To construct a *fur* deletion derivative, Δ*fur*, the primers Δfur_FF_xbaI and Δfur_FR_speI (Supplementary Table S2) were used for amplification of the upstream region of *fur*, and Δfur_BF_speI and Δfur_BR_xhoI for the downstream region of *fur*. The PCR products were cloned to the predigested suicide vector pDM4. The resulting plasmid was mobilized from S17-1 *λpir* to the wild type *V. vulnificus* MO6-24/O strain by conjugation. A double crossover was selected in LB plated with 10% sucrose. Colonies that grew on sucrose plate but sensitive to chloramphenicol were selected. The mutation was confirmed through PCR and DNA nucleotide sequencing.

### Bioluminescence assays

Derivatives of *V. vulnificus* MO6-24/O or its *fur*-deletion isotype, Δ*fur*, harboring the *luxAB* reporter transcriptionally fused to each of *leuO*, *vhuα*, *vhuβ*, *rpoS*, and *katG* were described previously (Kim et al., 2018). Overnight cultures of tested strains harboring the *luxAB* reporter fusions grown in LB were inoculated into fresh LB medium. To make an iron-limiting condition, 100 μM 2,2′-dipyridyl was added exogenously to the LB broth when the A_600_ value of the culture reached approximately 0.1, and samples were diluted 125-fold with LB broth. At various growth stages, 0.006% (v/v) n-decylaldehyde (in 50% ethanol) was added and luminescence was measured using a microplate reader (Mithras LB 940; Berthold, Bad Wildbach, Germany) as previously described (Wen et al., 2012). The specific transcription level was expressed as relative light units (RLU) normalized to cell density.

### Site-directed mutagenesis of putative Fur boxes in the upstream region of *leuO*

The 1,540-bp DNA fragment of the *leuO* upstream region (−975 to +565 with respect to the translation start site) was amplified by PCR using the primers leuO_DCO_F and leuO_DCO_R. The resulting product was ligated to the pGEM-T Easy vector to construct pGEM-LeuO. To introduce mutations into each of the four regions (SM1 ~ 4) containing putative Fur boxes, mutagenesis was performed using primer sets leuO_SDM_1_F and leuO_SDM_1_R for SM1, leuO_SDM_2_F and leuO_SDM_2_R for SM2, leuO_SDM_3_F and leuO_SDM_3_R for SM3, and leuO_SDM_4_F and leuO_SDM_4_R for SM4. The resulting four plasmids were named pGEM-SM1 through pGEM-SM4, respectively.

### Expression and purification of Fur

The Strep-tagged Fur was expressed in *E. coli* BL21(DE3) cells harboring the *fur* clone pASK-IBA7-Fur (Kim et al., 2013b) by induction with 0.2 μg/mL anhydrotetracycline. After centrifugation, bacterial pellets were resuspended in a buffer (100 mM Tris-Cl, 150 mM NaCl, and 1 mM EDTA, pH 7.5). The cells were then sonicated and centrifuged at 4,585 × *g* for 10 min. The resulting supernatant was subjected to purification using Strep-Tactin affinity resin (IBA BioTAGnology, Göttingen, Germany), and specifically bound protein was eluted with E buffer (100 mM Tris-Cl, 150 mM NaCl, and 1 mM EDTA, and 2.5 mM desthiobiotin, pH 7.5) according to the manufacturer’s instructions. The eluted protein was separated on a 12% SDS-PAGE to assess the purity. The purified Fur protein was dialyzed using Spectra/Por molecular porous membrane tubing (molecular weight cutoff of 10,000; Spectrum Laboratoried Inc., Rancho Dominguez, CA) with A buffer (50 mM Tris-Cl, 100 mM NaCl, 1 mM MgCl_2_, and 2 mM dithiothreitol, pH 8.0). The protein was concentrated using the Vivaspin 6 instrument (Vivagen, Seoul, Korea). The protein concentration was determined by the Bradford method (Bradford, 1976).

### Gel shift assay

To assess the binding of Fur to upstream region of *leuO*, the 299-bp regions (−288 to +11 with respect to the translation start site) of the gene containing wild type and mutated bases at each of Fur box candidates (SM1 ~ 4) were amplified by PCR with the primers leuO_EMSA_F and ^32^P-labeled leuO_EMSA_R using pGEM-leuO and pGEM-SM1 ~ 4 as template DNAs. Similarly, to assess the binding of Fur on the region upstream to *vhuα*, the 303-bp regions (−261 to +42 with respect to the translation start site) were amplified by PCR with the primers HU_alpha_EMSAF and ^32^P-labeled HU_alpha_EMSAB. In the same way, for *vhuβ* used the 257-bp regions (−212 to +45 with respect to the translation start site); for *rpoS*, the 650-bp regions (−556 to +94 with respect to the translation start site); and for *katG*, the 495-bp regions (−401 to +94 with respect to the translation start site) were amplified by PCR with the each primer set HU_beta_EMSAF and ^32^P-labeled HU_beta_EMSAB, rpoS_EMSA_longF and ^32^P-labeled rpoS_EMSA_R, katG_EMSA_F and ^32^P-labeled katG_EMSA_R, respectively.

For gel shift assays, 10 ng of the labeled probe was incubated with increasing amounts of purified Fur protein in a 20 μL reaction mixture in binding buffer (Alice et al., 2008) containing 10 mM Tris-borate (pH 7.5), 100 μg/mL bovine serum albumin, 5% (v/v) glycerol, 40 mM KCl, 1 mM MgCl_2_, and 1 μg poly(dI-dC). The reaction mixture was supplemented with either 1 mM MnCl_2_ or 1 mM EDTA for 30 min at 30°C. The resulting mixtures were resolved in a 6% neutral polyacrylamide gel. Each of the labeled probes (10 ng) was incubated with increasing amounts of purified Fur protein, and gel shift assays were performed as described above. The gels were exposed to a BAS_MP 2040s imaging plate (Fujifilm, Tokyo, Japan) and scanned using a BAS-1500 instrument (Fujifilm).

### DNase I foot-printing analysis

An end-labeled 405-bp DNA fragments of the *leuO* upstream region (−376 to +29 with respect to the translation start site) was amplified using the primers leuO_fp_F2 and 6-FAM labeled leuO_fp2_R_FAM. To determine the Fur binding site, 200 ng of the amplified *leuO* upstream region was incubated with purified Fur (2 μM) in 50 μL of binding buffer containing 10 mM Tris-borate (pH 7.5), 100 μg/mL bovine serum albumin, 5% (v/v) glycerol, 40 mM KCl, 1 mM MgCl_2_, 1 μg poly(dI-dC) for 30 min at 30°C. After incubation, 0.01 unit of DNase I (Promega, Madison, WI) was added, and the reaction mixture was incubated at 37°C for 1 min. The reaction was terminated by the addition of 5 μL of RQ1 DNase stop solution (Promega, Madison, WI), and inactivated at 65°C for 10 min. To precipitate the samples, 55 μL phenol-chloroform was added, and the mixture was precipitated at room temperature for 5 min. After centrifugation at 11,323 × *g* at 4°C for 5 min sodium acetate (NaCOOH, pH 5.2) was added to 50 μL of supernatant. Following the addition of 100 μL of 100% ethanol, the mixture was incubated for 1 h at −80°C and then centrifuged in 11,323 × *g* at 4°C for 15 min. The sample was then washed with 70% ethanol, dried in water bath at 60°C, and dissolved in 10 μL of distilled water. DNA sequencing of the sample was carried out in GBST (Green-Bio Science and Technology, Seoul National University). Raw data obtained through ABI 3730xl were analyzed using the Peak Scanner software (Applied Biosystems, Waltham, MA, United States).

### *β*-galactosidase assay

*β*-galactosidase activity was measured as described previously (Miller, 1972). Briefly, *V. vulnificus* strains were cultured overnight in LB medium, harvested, and diluted to an A_600_ of 0.005, and *β*-galactosidase activities from cells harboring the genes transcriptionally fused with *lacZ* as described above was measured.

### Quantitative real-time PCR (qRT-PCR) analysis

RNA was isolated from *V. vulnificus* using the easy-BLUE™ total RNA extraction Kit (iNtRON Biotechnology, Seongnam, Korea) and treated with the RNase-free DNase set (Promega, Madison, WI, United States) to remove any residual DNA. The purified RNA was quantified using a Biophotometer (Eppendorf, Hamburg, Germany). Subsequently, cDNA was synthesized from 500 ng of RNA using the CellScript^™^ All-in-One cDNA Master Mix (Cellsafe, Yongin, Korea) following the manufacturer’s instructions. One microliter of cDNA was used for RT-PCR analysis on a Stratagene Mx3000p qPCR machine (Agilent Technologies, Santa Clara, CA, United States) using QGreenBlue 2 × Green qPCR Master Mix (Cellsafe, Yongin, Korea). The RT-PCR reactions were performed in triplicate in a 96-well plate using primer shown in Supplementary Table S2. The PCR conditions used to amplify all genes were: 10 min at 95°C and 40 cycles of 95°C for 15 s and 64°C for 40 s. The genes encoding type I glyceraldehyde-3-phosphate dehydrogenase (RS_10395) and DNA-directed RNA polymerase subunit alpha (RS_13660) of *V. vulnificus* were used as endogenous loading controls. Quantification was carried out using the Light Cycle 480 II real-time PCR system software program.

### Catalase activity assay

*V. vulnificus* strains (MO6-24/O, Δ*fur*, Δ*fur* complemented with pRK415-*fur*, Δ*katG*) were cultured overnight in LB medium, washed, and sub-cultured in fresh LB medium. All cells were harvested at exponential phase (A_600_ value of approximately 1.0) and sonicated using Ultrasonic Homogenizer (KUS-650, KBT, Seongnam, Korea) in 10 cycles with 1 s of sonication followed by 2 s of rest for each cycle. After centrifugation, the supernatant was concentrated using Amicon Ultra-0.5 mL Centrifugal Filter Units (10,000 NMWL, UFC501024, Merck-Millipore, Germany). Each of 50 μL sample was mixed with 10 mM hydrogen peroxide (Duksan, Ansan, Korea). The reaction tubes were vortexed, incubated for 2 min at 37°C, and 600 μL of working solution was added. The working solution consisted of 100 mL cobalt (II) solution (20.3 g / 1 L DIW), 100 mL sodium hexametaphosphate solution (10 g / 1 L DIW), 800 mL sodium bicarbonate solution (180 g/ 2 L DIW) (Hadwan, 2018). The tubes were vortexed for 5 s and then kept at room temperature for 10 min in the dark. After 10 min, the catalase activities were measured at 440 nm using a Multimode Plate Reader (PerkinElmer, Waltham, MA, United States).

### Quantification of the cFP production from *Vibrio vulnificus* MO6-24/O by HPLC

The flow rate was set to 1.0 mL/min, and the cFP peak was detected at 256 nm. As a reference, a 1 mM cFP solution (from a company) dissolved in 30% methanol was also prepared and analyzed using the same HPLC method. The amount of cFP in each sample was estimated based on the area of the cFP peak observed in the 1 mM cFP reference sample.

After culturing cells in LB broth for 24 h, a 20 mL supernatant of each sample was collected and mixed with an equal volume of ethyl acetate, and then finally dissolved in 150 μL of 30% methanol. The cFP in each sample was resolved using high-performance liquid chromatography (HPLC) using a C_18_-reverse phase column Mightysil RP-18 GP (Kanto, Tokyo, Japan) with 30% methanol as the mobile phase. The flow rate was set to 1.0 mL/min, and the cFP peak was detected at 256 nm. As a reference, 1 mM of cFP (Bachem, Bubendorf, Switzerland) dissolved in 30% methanol were also prepared in the same way, and analyzed using the same HPLC methods. The amount of cFP in each sample was estimated based on the area of the cFP peak observed in the 1 mM cFP reference sample.

## Results

### Fur represses the transcription of *leuO*, the master regulator for the cFP-ToxR pathway

We initially examined the effect of Fur-iron on the transcription of *toxR* encoding inner membrane porin, which serves as receptor for cFP (Park et al., 2006, 2019). Using a *lacZ*-fusion, we quantitatively compared the transcription level of ToxR in wild type and Δ*fur* (*fur*-deletion isotype) strains in the presence or absence of iron. However, no significant difference was observed between two groups of cells (data not shown). Furthermore, through a gel-shift assay using 363-bp region upstream to *toxR* as a probe (Supplementary Figure S1A) and purified ToxR, we determined that Fur does not bind to the upstream region, regardless of iron availability (Supplementary Figure S1B). These results indicate that the expression of ToxR is not influenced by iron.

**Figure 1:**
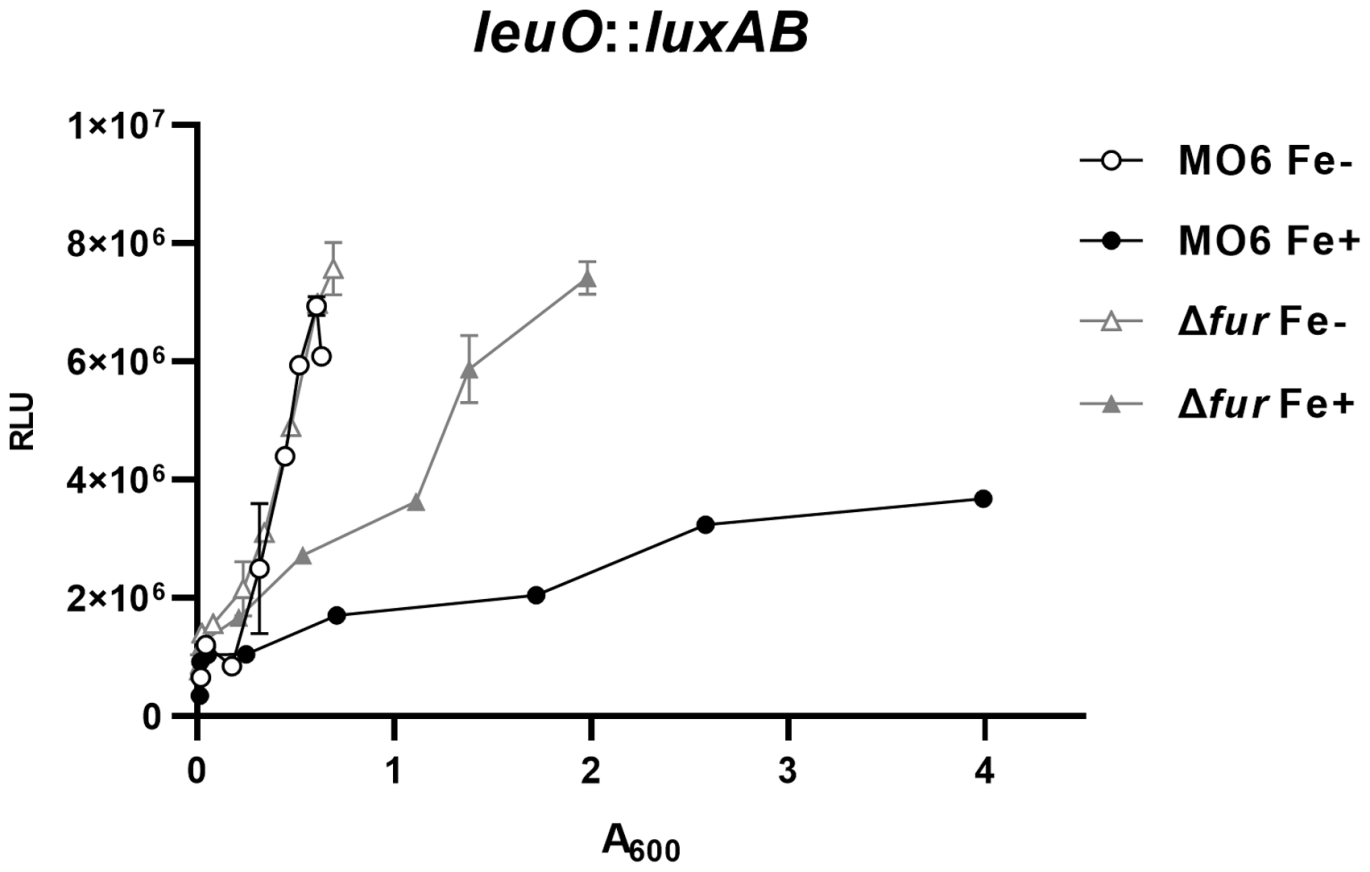
Fur-iron represses the transcription of *leuO*. The transcriptional activities of *leuO* were assessed using the *leuO*-*luxAB* reporter in wild-type *V. vulnificus* strain MO6-24/O (represented by circles) and the fur deletion mutant (Δ*fur*) (represented by triangles) under conditions of iron limitation (blank) or iron abundance (solid). Relative light units (RLU) were measured and normalized to cell density (A_600_). To induce iron limitation, 2,2′-dipyridyl (100 μM), an iron chelator, was added when the A_600_ reached 0.1.

In our previous study (Park et al., 2019), we investigated the regulatory elements in the region upstream to *leuO*, which encodes the master regulator for the cFP-signaling pathway in *V. vulnificus*. Within this region, we identified a putative Fur-binding sequence, suggesting that Fur may be involved in the regulation of *leuO* expression. To examine this possibility, we constructed the *luxAB* reporter gene fusions with *leuO* in both wild-type MO6-24/O strain and the Δ*fur*, and quantitatively measured luciferase activities in the presence or absence of iron. When iron was supplied, the RLU values in the Δ*fur* mutant were approximately three times higher than these in the wild-type strain (Figure 1). Under iron-limited conditions, there was no significant difference in *leuO* expression was observed between the wild-type strain and Δ*fur* mutant. It is noteworthy that, even in Δ*fur* cells, the expression of *leuO* was still repressed to some extent by iron, albeit significantly less compared to the wild type cells. This suggests the possible involvement of unidentified factor(s) in the iron-dependent regulation of *leuO*.

**Figure 2:**
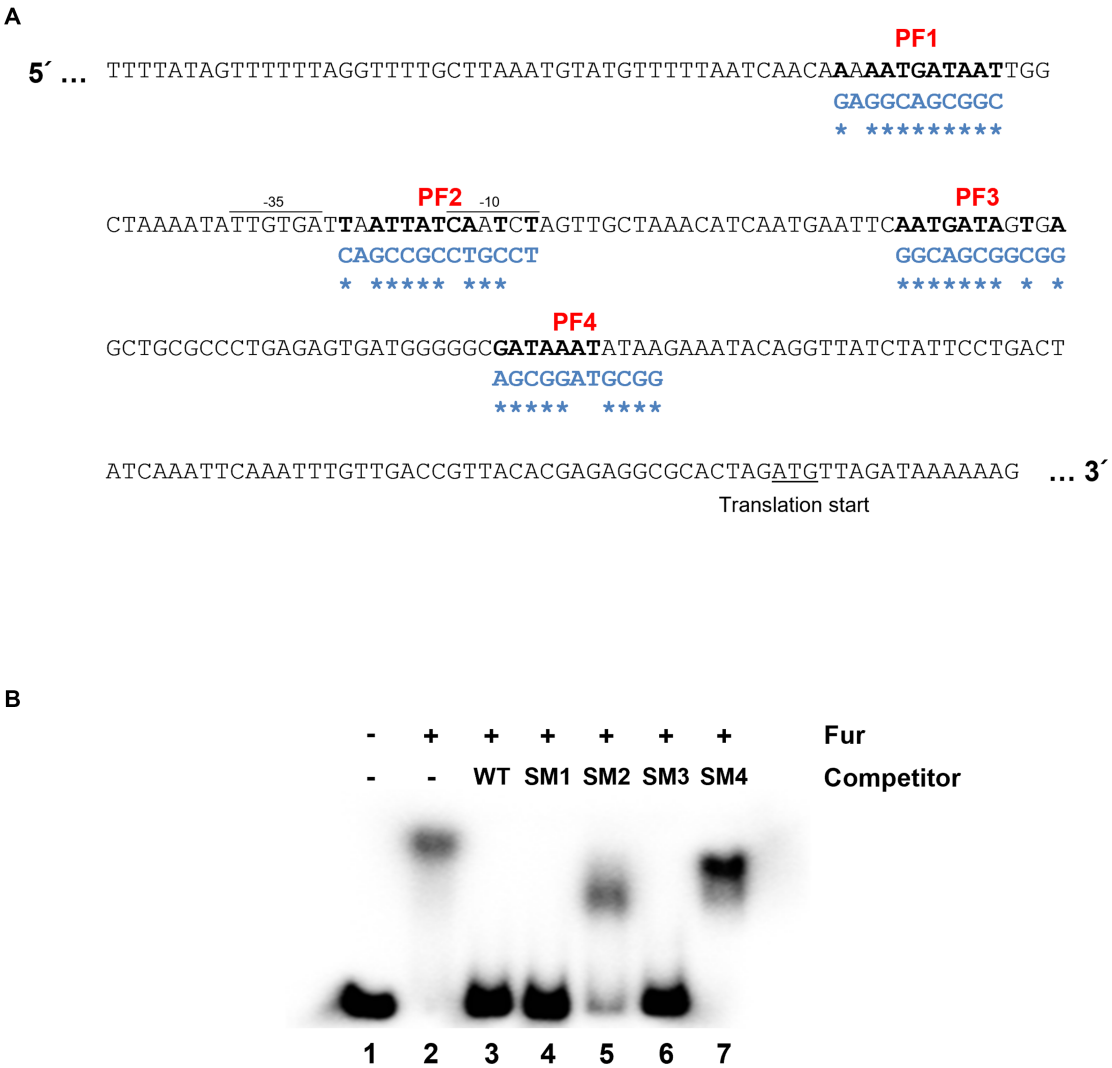
Fur-iron binding sites on the region upstream to *leuO*. **(A)** Nucleotide sequences upstream to *leuO*. The promoter region (−35 and −10 sites) and translation start site are indicated. Nucleotide sequences homologous to the Fur box (5′-GATAATGATAATCATTATC-3′) are named PF (putative Fur box) 1 through 4, and are denoted in bold. Nucleotide sequences of oligomers with site-directed mutations are denoted in blue letters, and the mutated sites are indicated with asterisks. **(B)** Gel shift assay indicates that Fur-iron binds to PF2 and PF4 regions. Shown here are the results of gel shift assays using the radiolabeled 299-bp fragment probe with the sequences of the region upstream to *leuO* and purified 200 nM Fur with 1 mM MnCl_2_. Lane 1 represents a negative control, which does not have any competitor or Fur, but only has 10 ng of the wild-type probe. Lane 2 has no competitor, but 200 nM Fur and 10 ng of the wild-type probe. Lanes 3 to 7 contain 200 nM Fur with 100 ng of the wild-type probe, and each of competitor SM1, SM2, SM3, and SM4, respectively, which are the 299-bp fragments with mutated sequences in PF1 through 4 regions.

The *leuO* gene encodes a key regulatory component in cFP – signaling pathway which is closely associated with virulence of pathogenic *Vibrio* species, and hence its regulation is important for the pathogenicity. Therefore, we defined in detail the *cis*-acting elements for Fur in the gene. There exist four regions with nucleotide sequences homologous to the Fur box (Wen et al., 2016) in the *leuO* upstream region, named PF (putative Fur box) sites 1 ~ 4 (Figure 2A). Meanwhile, gel shift assay demonstrated that purified Fur binds to the upstream region of *leuO* and it appears that at least two shifts occurred (Supplementary Figure S2A). To determine which sites of these putative Fur-binding sites are actually bound by Fur, we performed competition gel-shift assay using a radiolabeled 299-bp oligomer, as described in Materials and Methods, as a probe and purified Fur. The probe was synthesized by PCR using leuO_EMSA_F and leuO_EMSA_R as primers (Supplementary Table S2). We then prepared four DNA oligomers of the same size with mutations in each of those four PF sites (named SM1 ~ 4) as shown in Figure 2A. Each of these mutated oligomers, without radiolabeling, was added as a competitor at about 10-fold higher concentration than the radiolabeled probe oligomer. As shown in the lane 3 of Figure 2B, the competitor without any mutation completely outcompeted the binding of Fur for the probe. The competitor without any mutations in each of the PF1 and PF3 sites also hindered the binding to Fur to the probe (lanes 4 and 6). In contrast, the competitor oligomer with mutations in each of PF2 or PF4 sites did not significantly interfere in the binding of Fur onto the probe oligomer, while the competitor with mutations in PF2 slightly interfered in the binding, but much less than the one with mutation in PF4. In addition, footprinting experiments also show high binding affinity to the PF2 and PF4 regions (Supplementary Figure S2B). These results suggested that Fur strongly binds to the PF4 region, and weakly to the PF2 region.

### Transcription of another target gene set, *vhuαβ* modulated by LeuO also is repressed directly by Fur

**Figure 3:**
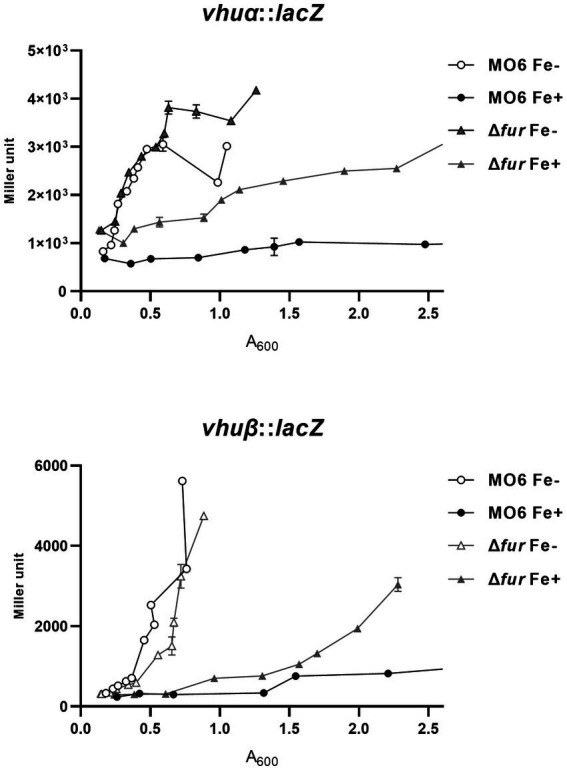
Transcriptional expression of *vhuα* and *vhuβ* also is repressed by Fur and iron. The transcription levels of *vhuα* and *vhuβ* were measured by *β*-galactosidase activities using *vhuα*-*lacZ* and *vhuβ*-*lacZ* transcription reporter in wild-type *V. vulnificus*, MO6-24/O (circles) and Δ*fur* (*fur* deletion mutant) (triangles) under iron-limiting (white symbols) or iron-rich (black symbols) conditions. When A_600_ reached 0.1, 100 μM 2,2′-dipyridyl was added as a chelator. Miller units represent the production of *β*-galactosidase values normalized to cell density. The error bars denote standard deviations of the results of three independent experiments.

In a previous study, it was demonstrated that LeuO has a negative regulatory effect on two genes, *vhuα* and *vhuβ*, which encode histone-like proteins that are involved in the regulation of numerous genes (Balandina et al., 2002; Grove, 2011; Kim et al., 2018). To investigate the potential regulation exerted by Fur-iron on these genes, we examined their transcriptional levels using *lacZ* transcriptional fusions in both wild-type and Δ*fur* cells under iron-rich and iron-depleted conditions (Figure 3). In wild-type cells, the transcription of both *vhuα* and *vhuβ* genes was repressed in the presence of iron. However, in the *fur*-deletion mutant cells, the expression of these genes was derepressed under iron-depleted conditions. In the presence of iron, the expression was still repressed, but to a significantly lesser extent than in wild-type cells. To further investigate the binding of Fur to the upstream regions of these two genes, we performed gel-shift assays using purified Fur in the presence or absence of iron. The results showed that Fur only binds to the upstream regions when iron is present (Supplementary Figure S3). These findings suggest that Fur-iron complex plays a role in the regulation of *vhuα* and *vhuβ* genes, and its binding is dependent on the availability of iron.

### Fur-iron represses the transcription of *rpoS*

**Figure 4:**
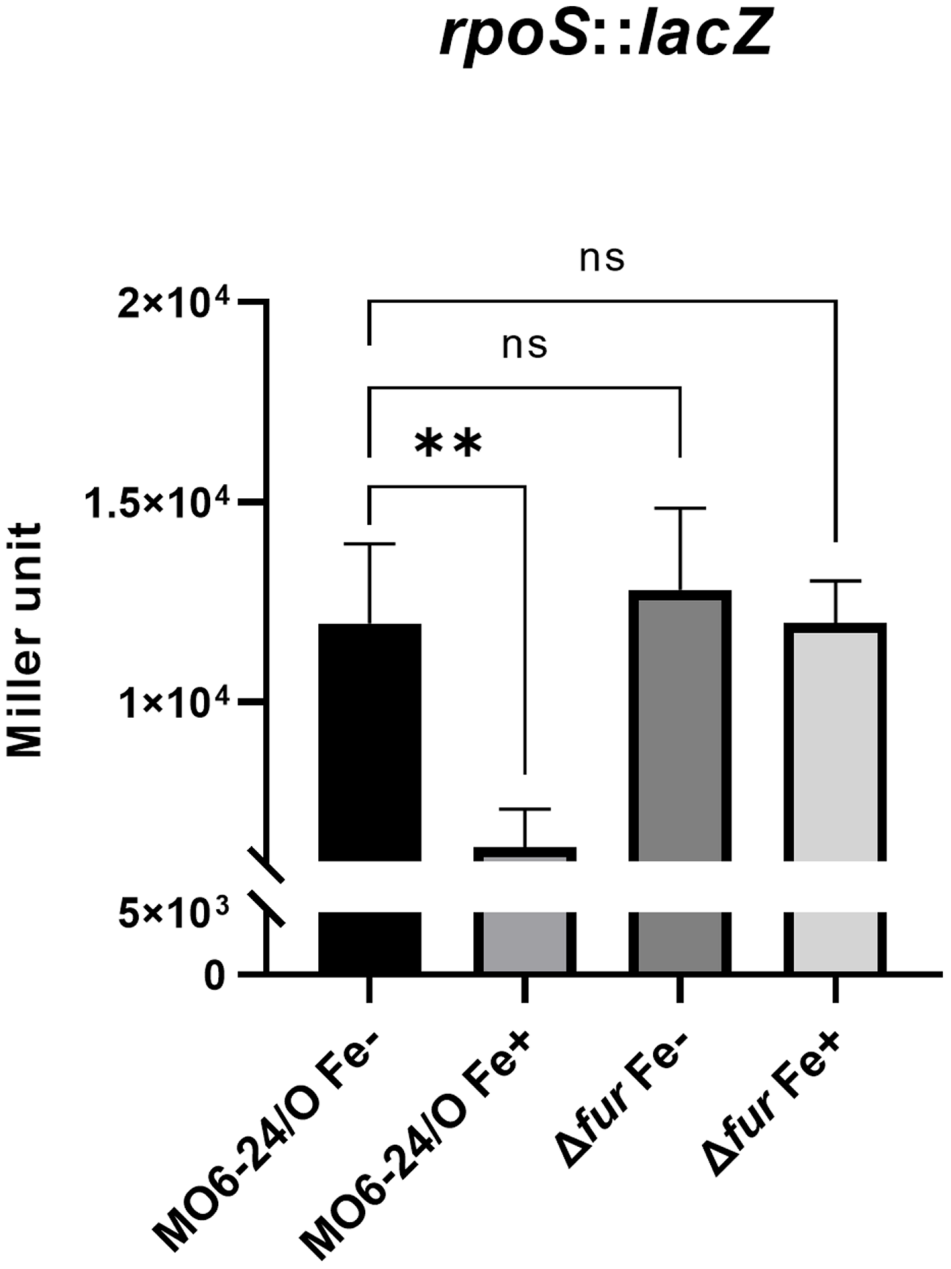
Fur-iron that represses transcription level of *rpoS*. The transcription level of *rpoS* were measured by *β*-galactosidase activities from *rpoS*-*lacZ* transcription reporter in wild-type *V. vulnificus*, MO6-24/O and Δ*fur* under iron-limiting (Fe-) or iron-rich (Fe+) conditions. Miller units represent the production of *β*-galactosidase values normalized to cell density. The data are average values from three independent experiments, and error bars denote the standard deviations indicated (Student’s *t*-test; **, 0.005 ≤ *p* < 0.05; ns, not significant).

We further investigated the impact of iron on the expression of *rpoS*, which encodes an alternative sigma factor and is involved in the cFP pathway. The activity of *β*-galactosidase, measured from a *lacZ* fusion to *rpoS*, was found to be strongly repressed by iron in wild-type cells. However, in the *fur*-deletion mutant, the activity was derepressed regardless of iron availability (Figure 4). To confirm the direct binding of Fur to the upstream region of *rpoS*, we performed a gel-shift assay, and the results are shown in Supplementary Figure S4.

### Fur-iron represses the transcription of *katG* encoding a catalase

**Figure 5:**
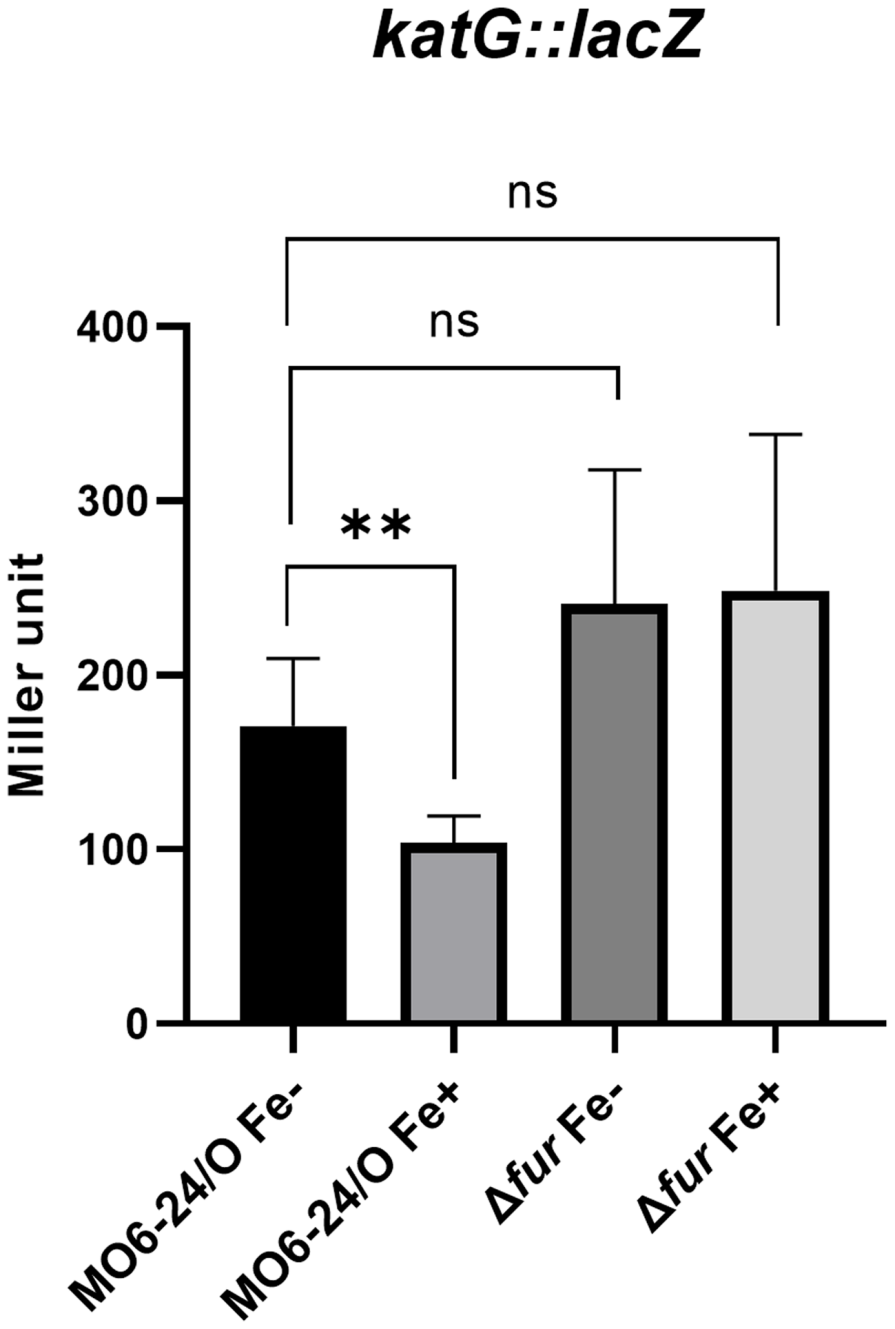
Fur-iron represses the catalase activity of *V. vulnificus* via regulating the transcription level of *katG*. The transcription level of *katG* were measured by *β*-galactosidase activities from *katG*-*lacZ* transcription reporter in wild-type *V. vulnificus*, MO6-24/O and Δ*fur* (*fur* deletion mutant) under iron-limiting or iron-rich conditions.

Our previous study demonstrated that *katG*, which encodes a catalase, is a member of the cFP-signaling regulon, and its transcription is regulated by RpoS (Kumamoto and Vukich, 1998; Kim et al., 2013b). Consequently, we investigated the direct regulation of the *katG* gene by Fur. To assess the impact of Fur on *katG* transcription, we utilized a *katG-lacZ* transcription fusion. The *β*-galactosidase activity from the reporter fusion in the Δ*fur* mutant was higher than that in the wild-type strain under iron-depleted conditions (Figure 5). In the *fur*-deletion mutant, the expression of *katG* was derepressed in the absence of iron; however, in the presence of iron, it remained repressed. Fur-iron repression occurs through direct binding to the upstream region of the gene (Supplementary Figure S5).

### Fur affects the expression of target genes in the RpoS regulon

**Figure 6:**
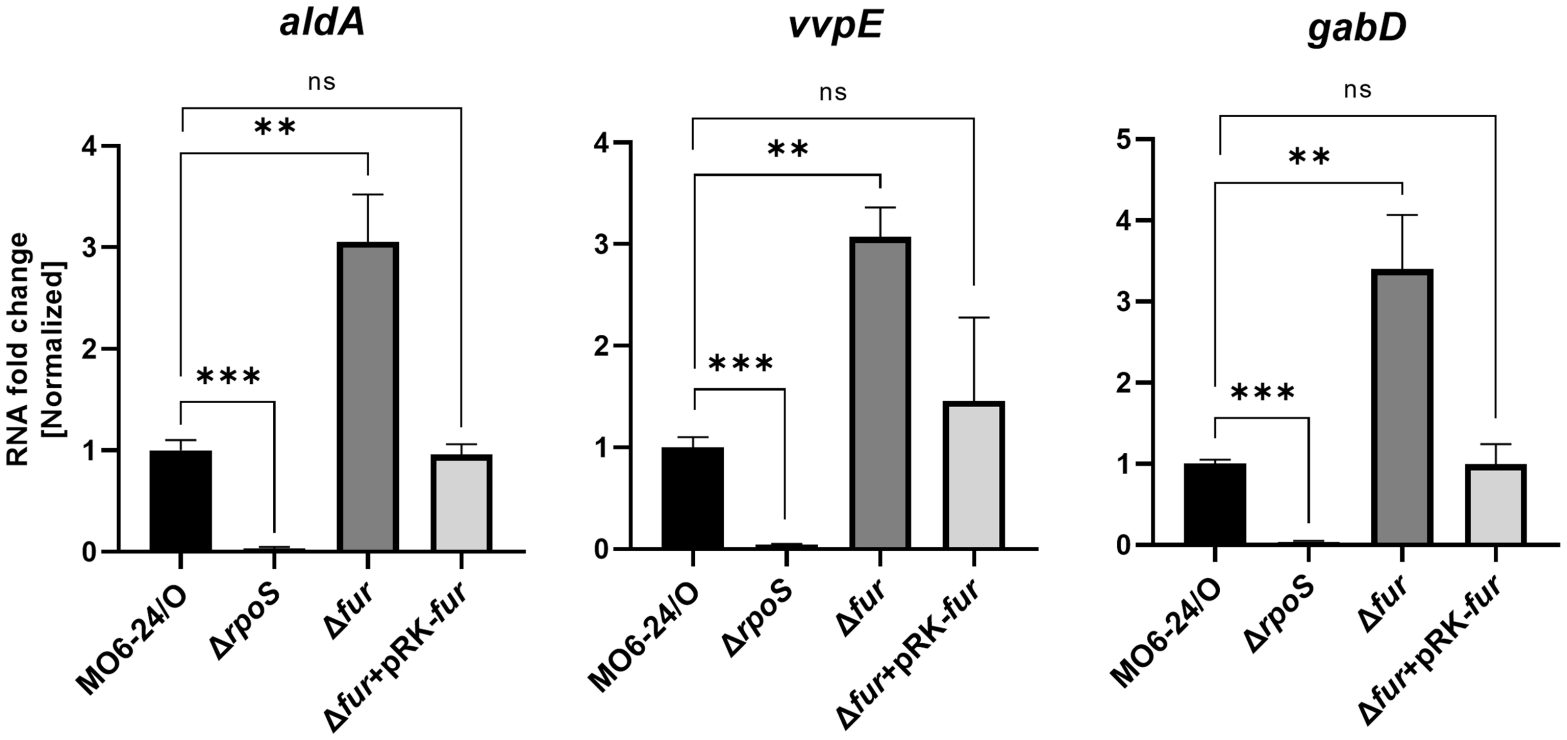
Transcription of the RpoS-inducing genes *aldA*, *vvpE*, and *gabD* is also repressed by Fur. The transcription levels of *aldA*, *vvpE*, and *gabD* in wild-type *V. vulnificus* MO6-24/O (pRK415), Δ*rpoS* (pRK415), Δ*fur* (pRK415), and Δfur (pRK-*fur*) cultured in LB broth as measured by qRT-PCR are shown. RNA levels were quantified using the comparative threshold cycle (ΔCt) method, and RNA-fold change was normalized to the value for MO6-24/O harboring pRK415. Values are averages from three independent experiments, and error bars denote standard deviations. The *p*-values for comparison with MO6-24/O are indicated (Student’s *t*-test; ***, 0.001 ≤ *p* < 0.005; **, 0.005 ≤ *p* < 0.05; ns, no significant).

RpoS is an alternative sigma factor known to initiate the transcription of numerous genes, forming a regulon. This suggests that these downstream genes may also be regulated by Fur-iron through modulation of RpoS expression. To investigate this, we measured the expression of three representative genes, *aldA* (encoding an aldehyde dehydrogenase), *vvpE* (encoding a metalloprotease), and *gabD* (encoding a succinate-semialdehyde dehydrogenase), known to be transcribed by RpoS in *V. vulnificus* (Jeong et al., 2001; Vijayakumar et al., 2004; Kim et al., 2018). The expression levels of these genes were semi-quantitatively measured using qRT-PCR. As we predicted, the results showed that all three genes were repressed by Fur in the presence of iron. In Δ*fur* isotype cells, the repression was relieved, and the introduction of exogenous *fur* in plasmid restored the repression (Figure 6). The nucleotide sequences in the regions upstream of these three genes did not show any apparent Fur box (Supplementary Figure S7), suggesting that the Fur-dependent repression of these genes was exerted via RpoS.

### Fur-iron does not affect the cFP production in *Vibrio vulnificus*

We expanded our investigation to examine the production of cFP, which serves as the signal molecule of the ToxR-LeuO-dependent QS system (Kim et al., 2018). To compare the levels of cFP in the culture supernatants, we employed HPLC. We found that the amounts of cFP detected in both wild-type cells and fur-deletion cells did not exhibit a significant difference (Supplementary Figure S6).

### Fur inhibits the catalase activity of *Vibrio vulnificus* in the presence of iron

**Figure 7:**
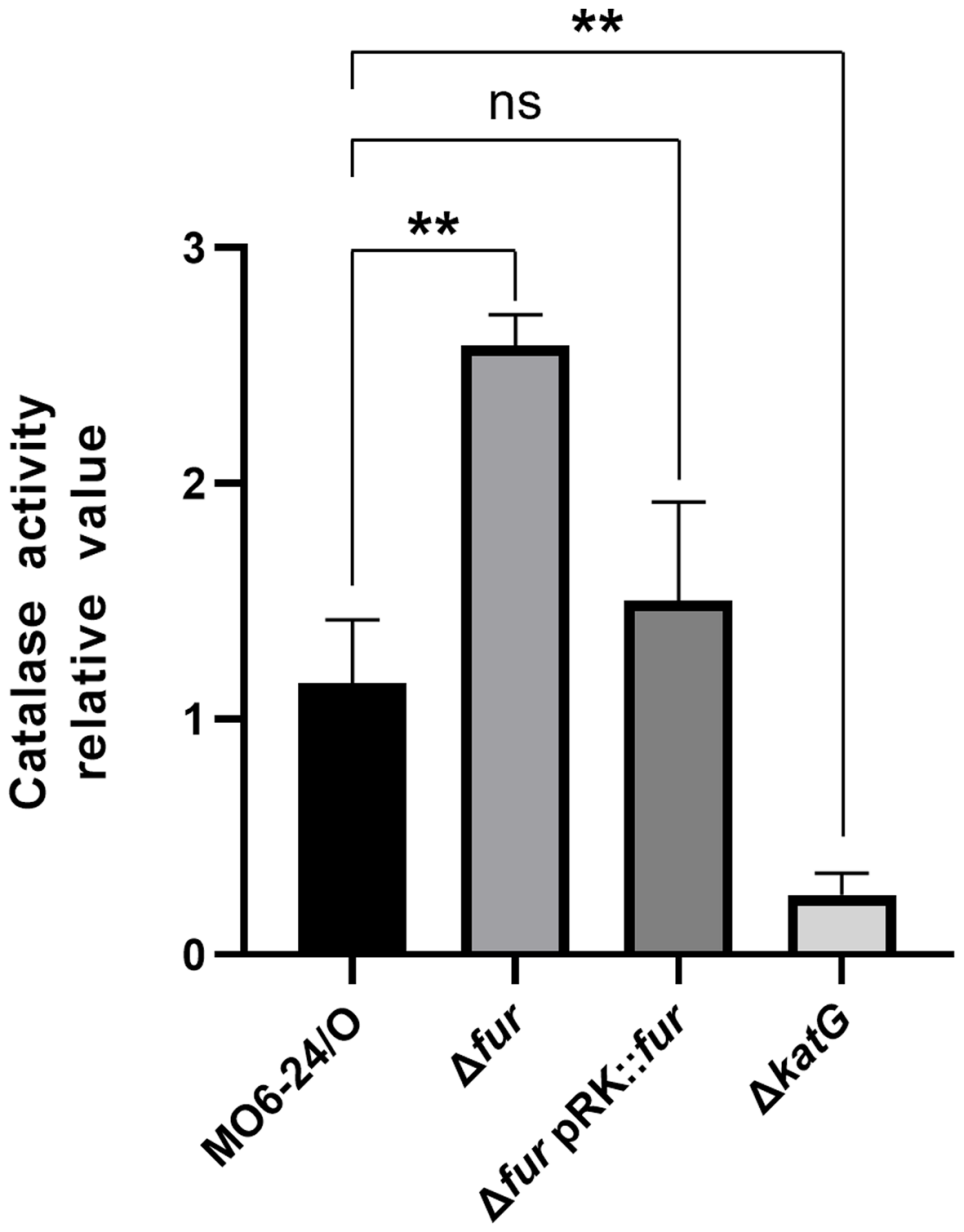
Fur-iron complex inhibits the catalase activity of *V. vulnificus*. Catalase activity was measured at 440 nm as described in the Materials and Methods section. Values are averages obtained from three independent experiments, and error bars denote standard deviations. The *p*-values for comparison with MO6-24/O under iron-limiting condition are indicated (Student’s *t*-test; **, 0.005 ≤ *p* < 0.05; ns, no significant).

The *katG* gene is a target virulence factor regulated by the cFP-mediated QS pathway. As shown in Figure 6, the transcription of *katG*, which encodes a catalase, is negatively controlled by Fur-iron. To quantitatively measure catalase activity, we performed assays on MO6-24/O, Δ*fur*, and Δ*fur*(pRK-*fur*) cells following the procedure outlined in the Materials and methods section. In Δ*fur* cells, a higher level of catalase activity was observed compared to wild-type cells. However, the introduction of the *fur* gene on a plasmid reduced the activity to the wild-type level (Figure 7). This finding is consistent with the results presented in Figure 5, suggesting that Fur represses the catalase activity of *V. vulnificus* by regulating *katG* expression.

### Fur–iron represses the expression of ToxR-dependent virulence factors of *Vibrio cholerae*

**Figure 8:**
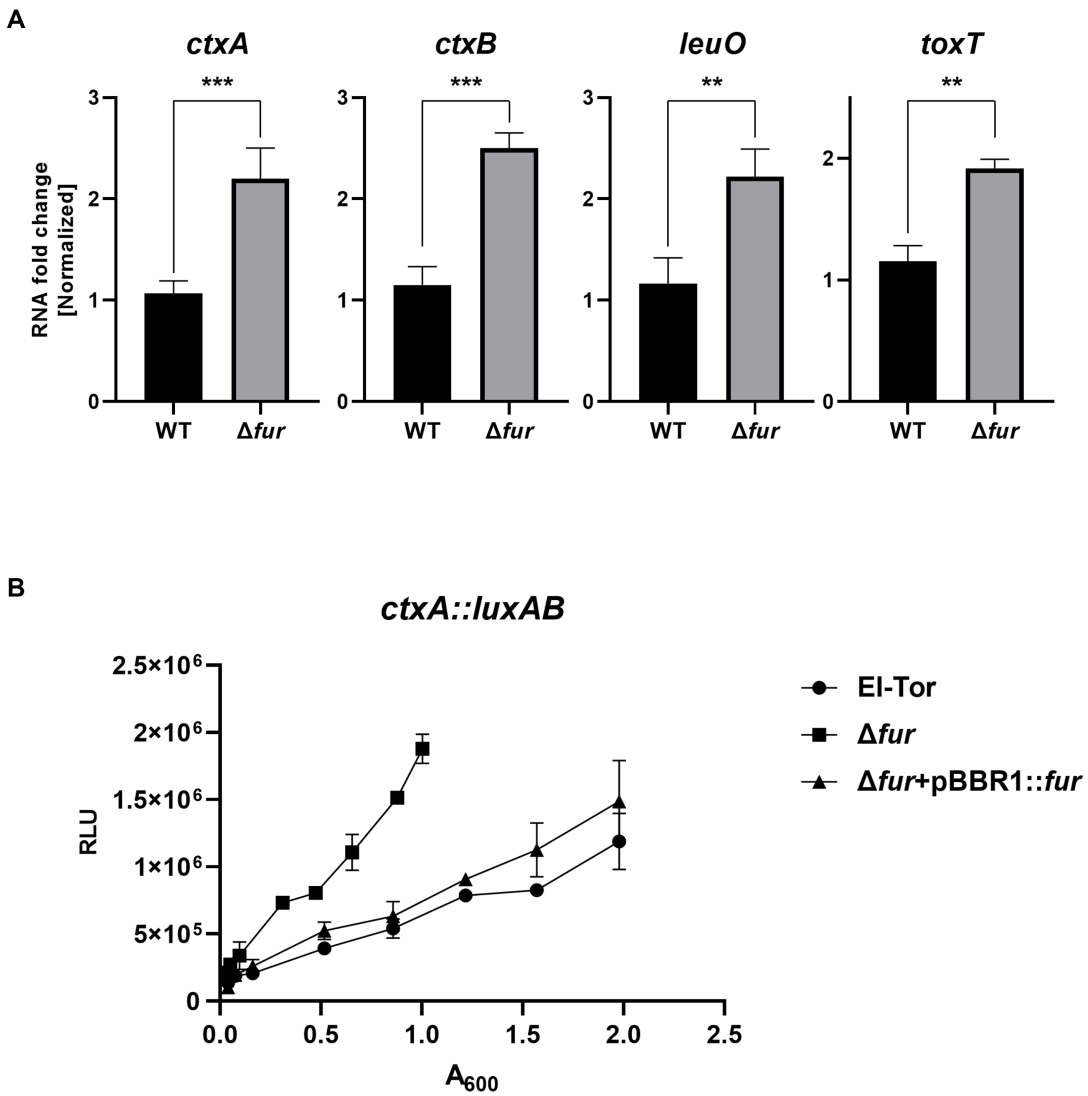
Fur-iron also inhibits *leuO* and virulence factors in *V. cholerae*. **(A)** The transcription levels of *ctxAB*, *leuO*, and *toxT* in *V. cholerae* (El Tor), and Δ*fur* cultured in LB broth as measured by qRT-PCR are shown. RNA samples obtained during the exponential phase were subjected to qRT-PCR analysis using the primers shown in Supplementary Table S2. The RNA levels were quantified using the comparative threshold cycle (ΔCt) method, and RNA-fold change was normalized to the value for El Tor. The *p*-values for comparison with El Tor are indicated (Student’s *t*-test; ***, 0.001 ≤ *p* < 0.005; **, 0.005 ≤ *p* < 0.05). **(B)** The transcriptional activities of *ctxA1* were assessed using the *ctxA1*-*luxAB* reporter in wild-type *V. cholerae*, El Tor (pBBR1-mcs2) (circles), Δ*fur* (pBBR1-mcs2) (squares) and Δ*fur* (pBBR1-*fur*) (triangles). Relative light units (RLU) represent the luminescence values, which were normalized to cell density (A_600_).

The cFP-mediated QS pathway is also present in the human pathogen *V. cholerae* (Park et al., 2006; Bina and Bina, 2010; Bina et al., 2013). In this pathogen, LeuO, which receives the cFP signal through ToxR similar to *V. vulnificus*, positively regulates the expression of *ctxAB*, the genes encoding the exotoxin cholera toxin, as well as ToxT, which induces the expression of various virulence-associated genes (Silva and Benitez, 2016; Bhandari et al., 2021; Bina and Bina, 2023). To explore whether Fur-iron also regulates these genes involved in the cFP pathway in *V. cholerae*, we compared the transcription levels of *leuO*, *toxT*, and *ctxAB* in the *V. cholerae* O1 El Tor strain N16961 and its *fur*-deletion derivative using qRT-PCR. As shown in Figure 8A, the expression levels of these four genes were significantly higher in the *fur*-deletion mutant compared to wild-type cells. Additionally, the transcription level of *ctxA*, which encodes the effector subunit of the cholera toxin, was quantitatively assessed using the *luxAB* reporter fusion (Figure 8B). Deletion of *fur* significantly enhanced *ctxA* transcription, and the introduction of the *fur* gene in trans complemented the mutant phenotype, confirming the repression of *ctx* by Fur.

## Discussion

Availability of iron ion is an important environmental factor affecting virulence of pathogenic microorganisms. We have studied QS signal transductions, which play important roles in regulating virulence factors, and various factors affecting the pathways, using pathogenic *Vibrio* species as model systems. Previous studies demonstrated that iron affects the AI-2-mediated QS signal pathway by regulating the expressions of components associated with the pathway (Wen et al., 2012, 2016; Lee et al., 2022). Iron is related to oxidative stress, and one of the target genes in the cFP-mediated QS signal pathway is *katG* encoding a catalase. Therefore, we extended our study to the cFP-mediated QS pathway. In this study, we found that iron also affects the expression of regulatory components in the cFP-mediated quorum-sensing pathway, and Fur is a major regulator responsible for the regulation, as is in the case of AI-2 QS.

All the cytoplasmic regulatory components of the cFP QS pathway including the master regulator LeuO, vHUα and β, and RpoS are repressed by Fur-iron, which directly binds to Fur boxes in the upstream region of each of those coding genes. Each of these cytoplasmic component also functions as a regulator forming its own regulon. Therefore, it was expected that the target genes of these regulators would also be indirectly regulated by Fur-iron. The role as a regulator of the alternative sigma factor RpoS has been well described. As such, we examined three target genes known to be downstream genes of RpoS; *aldA* (aldehyde dehydrogenase), *vvpE* (metalloprotease), and *gabD* (succinate-semialdehyde dehydrogenase). These genes, as expected, were regulated by iron in Fur-dependent manner, and the regulatory patterns were similar to that of RpoS. However, none of these three genes has a distinct Fur box near the promotes (data not shown), indicating that the Fur-dependent iron regulation is elicited via RpoS. Among these genes, *vvpE* has been well studied for its roles as virulence factor (Lee et al., 2016). This protease functions as an elastase that disrupts the tight junctions of human intestinal cells. The expression of *vvpE* is activated by SmcR, which is the master regulator of AI-2 QS system. Our previous study showed that expression of *smcR* is also directly repressed by Fur-iron (Kim et al., 2013b). Therefore, it is reasonable to postulate that VvpE, an elastase, contributes to release of iron from host human cells, and the expression of this enzyme is not necessary when iron is available. Unlike above three genes, which are transcribed by RpoS and their expressions are repressed by Fur-iron rather indirectly through RpoS, *katG* is directly repressed by Fur-iron. As shown in this study, the expression of this gene is also repressed by Fur-iron. The catalase activities from wild-type and *fur*-deletion isotype cells of *V. vulnificus* (Figure 7) also confirm this regulation. At the moment, we do not understand biological meaning of the tight repression of *katG* caused by Fur-iron. One possible scenario is that KatG may be defense mechanism required for the pathogen to protect itself against ROS produced by host cells. By the time infected host cells erupt and release iron, they may have lost the ability to produce ROS, and pathogen would not need KatG anymore. Therefore, the pathogen may tightly regulate the *katG* expression to save energy. Meanwhile three other RpoS-directed genes may have some other functions regardless of the presence of iron. Further study on biological functions of KatG would enable a more precise interpretation.

We observed that the signal molecule cFP is produced independently of iron. Functions responsible for the biosynthesis of cFP have not yet been elucidated in *Vibrio* spp., and therefore, we could not confirm this in a molecular genetic level, but HPLC analysis of cFP production clearly indicated that the product of cFP is not affected by either iron or Fur. Our previous study showed that the production of AI-2, which is a signal molecule for another QS pathway in *Vibrio* spp. is negatively controlled by the master regulator SmcR via repressing the expression of the small RNA RyhB, which is necessary for the full expression of LuxS, the AI-2 biosynthase (Lee et al., 2022). The expression of this small RNA is negatively controlled by Fur-iron. In other words, the expression of AI-2 signal molecule is negatively controlled by Fur-iron as is high cell density. This mechanism enables the feedback regulation of the AI-2-mediated QS pathway, and hence it is promptly shut off when cell density decreases, or when the environmental iron level is too high. Supplementary Figure S8 provides a schematic summary of the Fur-iron-mediated regulatory networks in QS pathways in *V. vulnificus*. These differences in regulation patterns between biosynthesis of AI-2 and cFP suggest that these two cognate signal pathways may have some different biological functions. It is possible that the AI-2 QS may be more closely related with cell density or iron level than the cFP QS. A clear explanation awaits identification of the functions of cFP biosynthesis and its regulatory aspects.

It is noteworthy that, in the *fur*-deletion mutant, expression levels of *leuO* and *vhuαβ* still appear to be modulated by iron. Expression levels of these genes in the *fur*-deletion mutant are much higher than in wild-type cells. Nevertheless, in the *fur*-deletion mutant, the expression is repressed significantly, even though it is still higher than in the wild-type cells (Figures 1, 3). This suggests that there is some unknown factor other than Fur involved in an iron-dependent regulation, and its effect is not as strong as that of Fur. One possible candidate responsible for the iron-dependent repression is the Csr pathway, which is reported to be involved in iron-dependent regulation of *toxR* in the related species *V. cholerae* (Mey et al., 2015). We recently found that this pathway is also functional in *V. vulnificus*, and that the expression is repressed by Fur-iron (unpublished data). Further study would clarify the connection between Csr and cFP QS pathways in terms of iron-dependent regulation.

Cells could repress the expression of all the downstream components just by repressing upstream regulatory components. However, our result apparently showed that Fur-iron directly represses each of the individual components of the downstream genes in the ToxR-dependent QS pathway to achieve a tight regulation in *V. vulnificus*. We cannot clearly rationalize this phenotype and it is possible that some ‘leaky’ expression of downstream genes may have inadvertent or disadvantageous effects on cells in the presence of iron. Alternatively, the main biological role of the cFP-ToxR dependent regulatory pathway of *V. vulnificus* may be closely associated with the availability of iron in host body, which is absolutely necessary for the survival of the pathogen. Hence, with iron available, the pathogenic cells may want to save energy by tightly shutting down those components. The pathogenic bacterium may have evolved such a modulation strategy to optimize its own physiology in response to iron levels in a host in various stages of infection. This study together with previous studies once again highlights the importance of iron in pathogenicity of virulent bacteria and the co-relationship between iron and QS regulation.

## Data availability statement

The original contributions presented in the study are included in the article/Supplementary material, further inquiries can be directed to the corresponding author.

## Author contributions

K-WL: Investigation, Methodology, Visualization, Writing – original draft. SK: Investigation, Writing – original draft. SL: Investigation, Writing – original draft. MK: Investigation, Writing – original draft. SS: Investigation, Writing – review & editing. K-SK: Conceptualization, Formal analysis, Funding acquisition, Project administration, Supervision, Validation, Writing – original draft, Writing – review & editing.

## Abbreviations

cFP: cyclic-phenylalanine-proline
QS: quorum sensing
AI-2: autoinducer-2
