# Supplementary material for: Iron-Fur complex suppresses the expression of components of the cyclo-(Phe-Pro)-signaling regulatory pathway in Vibrio vulnificus
Source: Front Microbiol. 2023 Oct 3;14:1273095. doi: 10.3389/fmicb.2023.1273095 (PMC10584307; doi:10.3389/fmicb.2023.1273095)
Supplement: Supplementary file 1 [file Data_Sheet_1.pdf]

Supplementary Material-1

Iron-Fur Complex Suppresses the Expression of Components of the Cyclo-(Phe-Pro)-Signaling Regulatory Pathway in *Vibrio vulnificus*

Keun-Woo Lee[1], Soyee Kim[1], Sora Lee[1], Minjeong Kim[1], Suji Song[1], and Kun-Soo Kim[1,2*]

*** Correspondence:** Kun-Soo Kim: kskim@sogang.ac.kr

Supplementary Tables

Supplementary Table 1. Strains or plasmids used in this study

Strains or plasmids	Derivation / relevant characteristics	Reference or source
**Strains**		
E. coli		
DH5α	λ^- φ80d*lacZ* Δ*M15* Δ(*lacZYA- argF*)U169 *recA1 endA1 hsdR17* ($r_K^- m_K^-$) *supE44 thi-1 gyrA relA1*	Our collection
S17-1	[C600::RP4-2 (Tc::Mu)(Km::Tn7) *thi pro hsdRM*$^+$ *recA*, Tp^r	(Simon et al., 1983)
S17-1 *λpir*	S17-1 with *λpir* lysogen	(Simon et al., 1983)
BL21(DE3)	F^- *ompT hsdSB* ($r_B^- m_B^-$) *gal dcm* (DE3)	Novagen
V. vulnificus		
MO6-24/O	Pathogenic clinical isolate	(Reddy et al., 1992)
Δ*leuO*	Derivative of MO6-24/O with a deletion in *leuO*	(Kim et al., 2009)
Δ*fur*	Derivative of MO6-24/O with a deletion in *fur*	This study
Δ*fur*Δ*leuO*	Derivative of MO6-24/O with a deletion in *fur* and *leuO*	This study
Δ*fur*Δ*vhuαβ*	Derivative of MO6-24/O with a deletion in *fur* and *vhuαβ*	This study
Δ*fur*Δ*rpoS*	Derivative of MO6-24/O with a deletion in *fur* and *rpoS*	This study
**Plasmids**		
pDM4	Suicide vector for allelic exchange, *sacB*, Cm^r	(Milton et al., 1996)
pGem-T Easy	TA-cloning vector, *lacZ*, f1 origin, Ap^r	Promega
All in One	TA-cloning vector, *lacZ*, f1 origin, Ap^r	Biofact
pRK415	IncP *ori*, broad-host-range vector; *oriT* of RP4, Tc^r	(Keen et al., 1988)

Supplementary Table 1. Strains or plasmids used in this study (continued)

Strains or plasmids	Derivation / relevant characteristics	Reference or source
pRK-*fur*	pRK415 with fur operon to complementation	(Kim et al., 2013)
pMZtc	pDM4 with the promoter-less lacZ gene for transcriptional fusion	(Park et al., 2006)
pMZtc-*toxR*	pMZtc with the promoter region of *toxR*	This study
pMZtc-*vhuα*	pMZtc with the promoter region of *vhuα*	(Kim et al., 2018)
pMZtc-*vhuβ*	pMZtc with the promoter region of *vhuβ*	(Kim et al., 2018)
pMZtc-*rpoS*	pMZtc with the promoter region of *rpoS*	(Kim et al., 2018)
pMZtc-*katG*	pMZtc with the promoter region of *katG*	(Kim et al., 2018)
pRKΩ*lacZ*	pRK with the promoter-less *lacZ* gene for transcriptional fusion	(Park et al., 2006)
pBBR1-MCS2	Broad range cloning vector, Km^r	(Kovach et al., 1995)
pBBR12-*leuO*	pBBR1-MCS2 with the *leuO* operon of *V. vulnificus*	(Kim et al., 2018)
pBBR12-*ompU*	pBBR1-MCS2 with the *ompU* operon of *V. vulnificus*	This study
pASK-IBA7-Fur	pASK-IBA7 containing *V. vulnificus* Fur	(Kim et al., 2013)
pHK0011	pRK415 with a promoterless *luxAB*, Tc^r	(Jeong et al., 2001)
pET28a-LeuO	pET28a containing *V. vulnificus* LeuO	(Park et al., 2019)

Supplementary Table 2. Primers used in this study

Name	Nucleotide sequence (5′ to 3′)
**Cloning of *V. vulnificus fur***	
Δfur_FF_xbaI	TCTAGAGACTTCTTCTCGATATTG
Δfur_FR_speI	ACTAGTAGCATCCTTTAGCGCTTG
Δfur_BF_speI	ACTAGTGACGCACATAAACGTAAG
Δfur_BR_xhoI	CTCGAGTAACCATCCAAATAAGCC
**Gel shift assay**	
leuO_EMSA_F	GGGTTAAGAGATGAGATATC
leuO_EMSA_R	TTATCTAACATCTAGTGCGC
smcR_EMSA_F	TGTTTTATCGTTGCTAATGG
smcR _EMSA_R	CCATAGGTTGTTTCCTTACC
HU_alpha_EMSAF	TGCCCTTGAAGAAATCCCTC
HU_alpha_EMSAB	TGCTTTCTCTGCGATAAAGTC
HU_alpha_B_FAM	FAM-TGCTTTCTCTGCGATAAAGTC
HU_beta_EMSAF	GCGTTTGACGCTAAAAAATAG
HU_beta_EMSAB	ATCTGCGTTTGCAGCGATTG
HU_beta_B_FAM	FAM-ATCTGCGTTTGCAGCGATTG
rpoS_EMSA_longF	AAGATCCAGTTAAGGGTA
rpoS_EMSA_R	CTTTTTCATTAATCTCGATAT
katG_EMSA_F	TCAACGCATTTAGTTG
katG_EMSA_R	TTGGCCACCAAGCGACAT
vc_ctxA_EMSA_F	TGGTGTTCGATACCTTTGCA
vc_ctxA_EMSA_R	TCATCAGGAGGTCTAGAATC
vc_toxT_EMSA_F	GTGGTGTGAAACTGTATAGC
vc_toxT_EMSA_R	CCAGAACATTTTGTAGTCGT
vc_leuO_EMSA_F	CCCATTAAAAATGCATTTTTA
vc_leuO_EMSA_R	ACTCATTGCGTCTTTTTTATCTA
ToxR_EMSA_F	TTATTGGAATACAGTGAG
ToxR_EMSA_R	TGCGATTACAAACTTAGT

Supplementary Table 2. Primers used in this study (continued)

Name	Nucleotide sequence (5′ to 3′)
**Site-directed mutagenesis**	
leuO_DCO_F	TCTAGATGCGTCATTTTGCTCCTGCT
leuO_DCO_R	CTCGAGCCACGAGTTCATCTTTGAAG
leuO_SDM_1_F	GAGGCAGCGGCTGGCTAAAATATTGTGATTA
leuO_SDM_1_R	GCCGCTGCCTCTGTTGATTAAAAACATACAT
leuO_SDM_2_F	TCAGCGGCCAATCTAGTTGCTAAACATC
leuO_SDM_2_R	GCGGCTGATCACAATATTTTAGCCAATT
leuO_SDM_3_F	GGCAGCGGCGGGCTGCGCCCTGAGAGTGATG
leuO_SDM_3_R	CCGCCGCTGCCGAATTCATTGATGTTTAGCA
SDM1_R	AGCCAGCCGCTGCCTCTGTT
SDM_WT1_R	AGCCAATTATCATTTTTGTT
SDM2_R	AGATTGGCGGCTGATCACAA
SDM_WT2_R	AGATTGATAATTAATCACAA
SDM3_R	CCGCCGCTGCCGAATTCATT
SDM_WT3_R	TCACTATCATTGAATTCATT
EMSA_SDM4_F	AGCGGATGCGGGAAATACAGGTTATCTATTC
EMSA_SDM4_R	CCGCATCCGCTGCCCCCATCACTCTCAGGGC
EMSA_SDM4_sR	CCGCATCCGCTGCCCCCATC
**Footprinting**	
leuO_fp_F	CAAAGGTGAGTGGTCATGTC
leuO_fp_R	CTCACTGCAGGCTGTGACA
leuO_fp_F2	CCCAGTTACGTGTCAATTTC
leuO_fp_R2	GCGCTCATCGCATCTTTTTT
leuO_fp2_R_FAM	FAM- GCGCTCATCGCATCTTTTTT
**Real time PCR**	
RpoS_RT_PCR_F	TACGCCGATTGGTGGAGATG
RpoS_RT_PCR_R	TCTCTTGCCCAACCTCTTCC
KatG_RT_PCR_F	GGTGGCCAAAGGCCCTCAAT
gapdh_qRT_F	CGTATCGGTCGTTTCGTTTT
gapdh_qRT_R	TACGTCAACACCGATTGCAT

Supplementary Table 2. Primers used in this study (continued)

Name	Nucleotide sequence (5′ to 3′)
RpoA_rtpcr_con_F	GTGACATCACCCATGACGGT
RpoA_rtpcr_con_R	TGCGATCTCTGCGTTGTCAT
RpoS_RT_PCR_F	TACGCCGATTGGTGGAGATG
RpoS_RT_PCR_R	TCTCTTGCCCAACCTCTTCC
KatG_RT_PCR_F	GGTGGCCAAAGGCCCTCAAT
KatG_RT_PCR_R	GTGCCACGCCATACGGATCA
gapdh_qRT_F	CGTATCGGTCGTTTCGTTTT
gapdh_qRT_R	TACGTCAACACCGATTGCAT
RpoA_rtpcr_con_F	GTGACATCACCCATGACGGT
RpoA_rtpcr_con_R	TGCGATCTCTGCGTTGTCAT
aldA_RTF	ATTGCCAAACTGGCGTTTAC
aldA_RTB	GTACACACTTCGCCCTGGTT
vvpE_RTF	TGTCCAGGAGCCAGCAATTA
vvpE_RTB	ACCATCGCCAAATGTCATCG
gabD_RTF	CCCGTAGGCAAACTGTTGAT
gabD_RTB	AACATAAAGGCGGTTTGCAC
vc_recA_RT_2_F	TCTTCGGCCCTGAATCTTCG
vc_recA_RT_2_R	CTGGCTGAGAAACCAGTAGC
vc_ctxA_RT_F	ACGGGATTTGTTAGGCACGA
vc_ctxA_RT_R	GGATGGGACTGTATGCCCC
vc_ctxB_RT_F	GTGCAGAATACCACAACACACA
vc_ctxB_RT_R	GCAATCCTCAGGGTATCCTTCA
vc_leuO_RT_F	CACGGCCGAACAATTGAGTG
vc_leuO_RT_R	CCAGTTCAGACTGACCGACC
vc_toxT_RT_2_F	GTAATTGGCGTTGGGCAGAT
vc_toxT_RT_2_R	CGCTAGCAAACCCAGACTGA

References for Supplementary Tables

Jeong, H. S., Jeong, K. C., Choi, H. K., Park, K.-J., Lee, K.-H., Rhee, J. H., et al. (2001). Differential expression of *Vibrio vulnificus* elastase gene in a growth phase-dependent manner by two different types of promoters. *J. Biol. Chem.* 276, 13875–13880. doi: 10.1074/jbc.M010567200.

Keen, N. T., Tamaki, S., Kobayashi, D., and Trollinger, D. (1988). Improved broad-host-range plasmids for DNA cloning in gram-negative bacteria. *Gene* 70, 191–197. doi: 10.1016/0378-1119(88)90117-5.

Kim, C.-M., Chung, Y.-Y., and Shin, S.-H. (2009). Iron differentially regulates gene expression and extracellular secretion of *Vibrio vulnificus* cytolysin-hemolysin. *J. Infect. Dis.* 200, 582–589. doi: 10.1086/600869.

Kim, I. H., Kim, S.-Y., Park, N.-Y., Wen, Y., Lee, K.-W., Yoon, S.-Y., et al. (2018). Cyclo-(L-Phe-L-Pro), a quorum-sensing signal of *Vibrio vulnificus*, induces expression of hydroperoxidase through a ToxR-LeuO-HU-RpoS signaling pathway to confer resistance against oxidative stress. *Infect. Immun.* 86, e00932-01017. doi: 10.1128/IAI.00932-17.

Kim, I. H., Wen, Y., Son, J.-S., Lee, K.-H., and Kim, K.-S. (2013). The Fur-iron complex modulates expression of the quorum-sensing master regulator, SmcR, to control expression of virulence factors in *Vibrio vulnificus*. *Infect. Immun.* 81, 2888–2898. doi: 10.1128/IAI.00375-13.

Kovach, M. E., Elzer, P. H., Hill, D. S., Robertson, G. T., Farris, M. A., Roop II, R. M., et al. (1995). Four new derivatives of the broad-host-range cloning vector pBBR1MCS, carrying different antibiotic-resistance cassettes. *Gene* 166, 175–176. doi: 10.1016/0378-1119(95)00584-1.

Milton, D. L., O'Toole, R., Hörstedt, P., and Wolf-Watz, H. (1996). Flagellin A is essential for the virulence of *Vibrio anguillarum*. *J. Bacteriol.* 178, 1310–1319. doi: 10.1128/jb.178.5.1310-1319.1996.

Park, D.-K., Lee, K.-E., Baek, C.-H., Kim, I. H., Kwon, J.-H., Lee, W. K., et al. (2006). Cyclo(Phe-Pro) modulates the expression of *ompU* in *Vibrio* spp. *J. Bacteriol.* 188, 2214–2221. doi: 10.1128/JB.188.6.2214-2221.2006.

Park, N.-Y., Kim, I. H., Wen, Y., Lee, K.-W., Lee, S., Kim, J. A., et al. (2019). Multi-factor regulation of the master modulator LeuO for the cyclic-(Phe-Pro) signaling pathway in *Vibrio vulnificus*. *Sci. Rep.* 9, 20135. doi: 10.1038/s41598-019-56855-4.

Reddy, G. P., Hayat, U., Abeygunawardana, C., Fox, C., Wright, A. C., Maneval, D. R., et al. (1992). Purification and determination of the structure of capsular polysaccharide of *Vibrio vulnificus* MO6-24. *J. Bacteriol.* 174, 2620–2630. doi: 10.1128/jb.174.8.2620-2630.1992.

Simon, R., Priefer, U., and Pühler, A. (1983). A broad host range mobilization system for *in vivo* genetic engineering: transposon mutagenesis in gram negative bacteria. *Nat. Biotechnol.* 1, 784–791. doi: 10.1038/nbt1183-784.

Supplementary Figures

a

Forward Primer

TTATTGGAATACAGTGAGTGGATCATCAGATGGAGAAGTTGTTTTACCTCTGAC

TGAAAGCCACGAGTTTCTTTATTATTTGCTACGGTCTCGTTCATTTTCACTCCAA

AGTTCTCTAATTGGGTGGGAAATCAATCGCTTGTTACGTAACATATGGCTGACA

-35

AATGTAAATTCAAGGTCAATTGTGGTAAAAACGCTGTTTT**TTTAGA**TAAATTT

-10

AATTATCCTATTGAGC**TAAATA**CATCTAAAGAAGCATAAGAGATGTTGGTCTA

AGCGCATATTGCGCGAATAAGCACCAAAAAACGCTGAATCATCGATATAAAGA

SD

AGAACCTCATGAGTAATATCGGCACTAAGTTTGTAATCGCA

Reverse Primer binding site

b

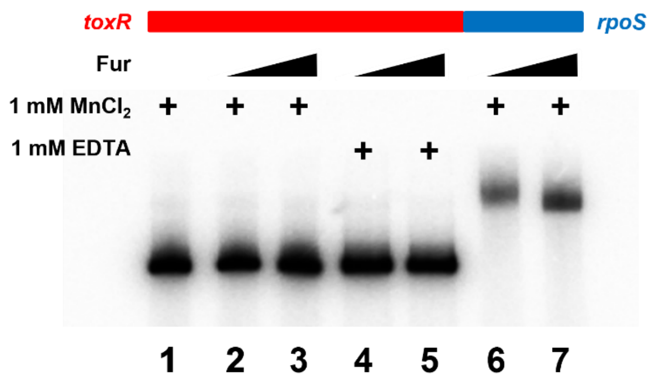

Supplementary Figure S1. Gel-shift assay showed that Fur fails binding to the upstream region of *toxR*.

(a) The nucleotide sequences upstream to *toxR*. Primer binding sites for the amplification to prepare DNA probe are indicated. Promoter and Shine-Dalgarno (SD) sequences are also marked. Sequences showing partial homology with Fur box are underlined. The coding sequences for *toxR* are highlighted in red. (b) The gel shift assay using purified Fur and a ^{32}P-labeled 363-bp DNA fragment of the upstream region of *toxR*. The Fur concentration used in lanes 2, 4, and 6 was 100 nM, and 200 nM in lanes 3, 5, and 7. Lanes 1, 2, 3, 6, and 7 were treated with 1 mM $MnCl_2$ to mimic the iron-rich environment, and lanes 4 and 5 were treated with 1 mM EDTA. In lanes 6 and 7 included the gel-shift assay of *rpoS* DNA with Fur serving as positive control (refer to Supplementary Figure S4). The electrophoretic mobility assay (EMSA) experiments were conducted as described in the Materials and Methods section.

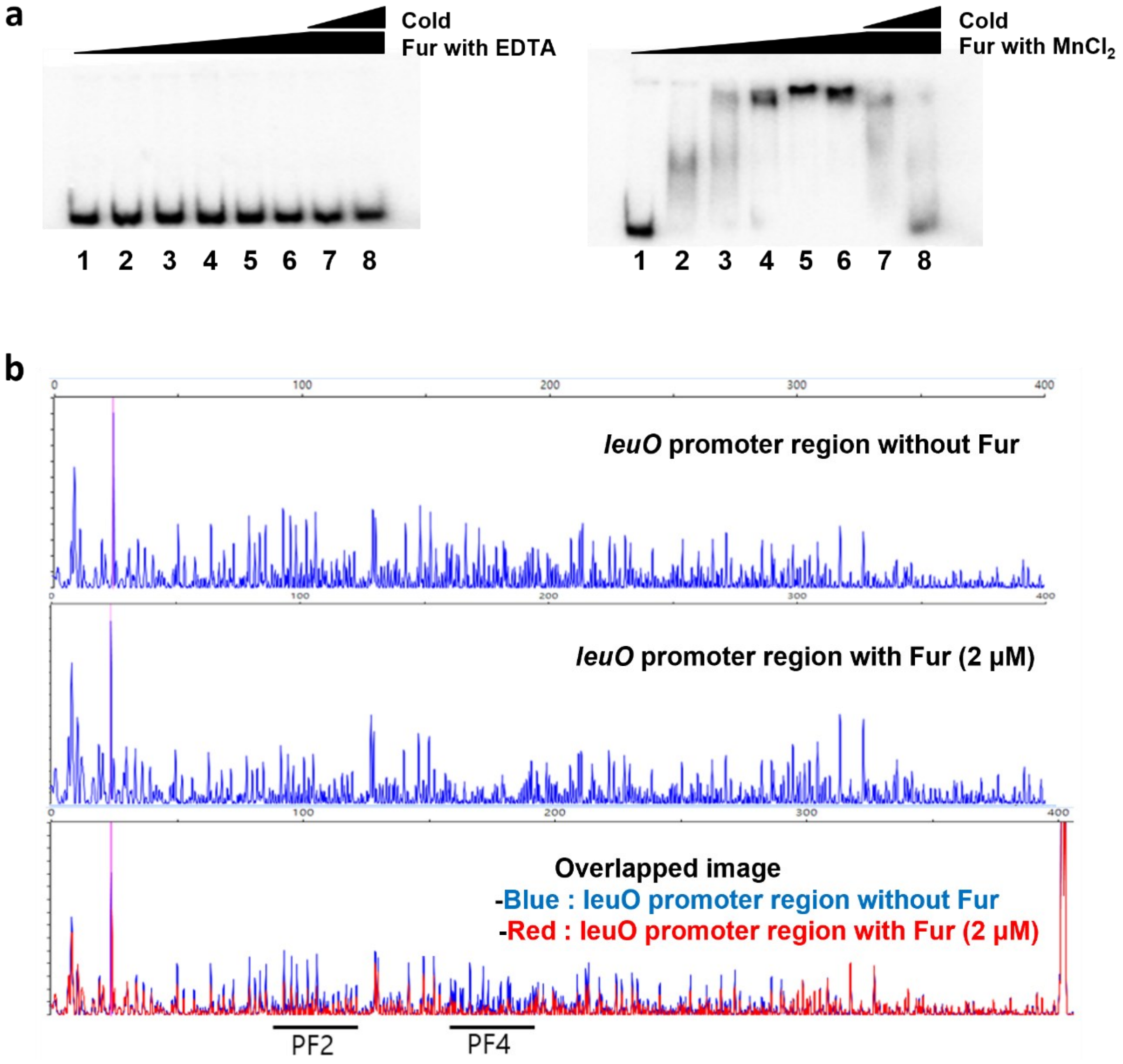

Supplementary Figure S2. Direct binding of Fur to the upstream region of *leuO* revealed by gel-shift assay and DNase I protection assay.

(a) Gel-shift assay using the radiolabeled 299-bp fragment from upstream *leuO* and purified Fur with either 1mM EDTA (left) or $MnCl_2$ (right). Lanes 1 to 5 represent increasing concentrations of Fur (0, 25, 50, 100, and 200 nM, respectively); while lanes 6 to 8 represent 200 nM Fur with unlabeled probes as competitors at 1, 10, and 100 ng, respectively. (b) DNase I protection assay showing region of the upstream of *leuO* protected from DNaseI digested Fur. The protected regions, designated as PF2 and PF4 (as described in the Result section), are indicated. The DNA used in the assay was 6-carboxyfluorescein (6-FAM)-labeled DNA.

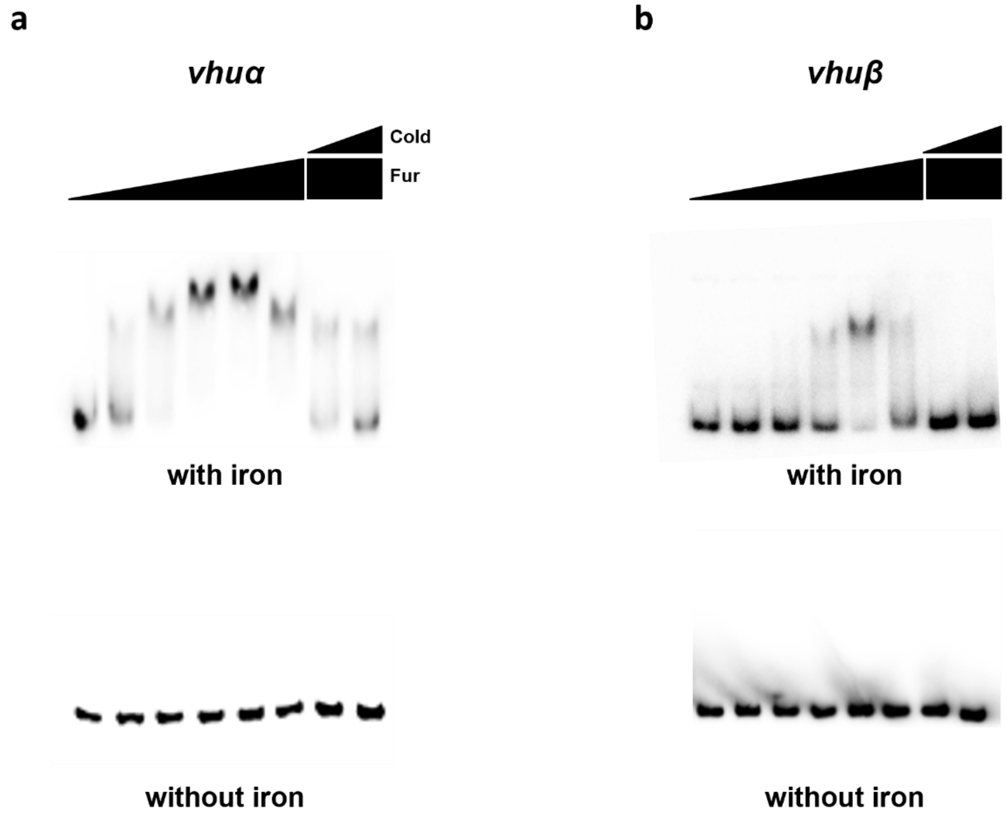

Supplementary Figure S3. Fur binding to the upstream regions of *vhuα* and *vhuβ*.

Gel shift assays were performed using 32P-labeled DNA fragments of the upstream region of *vhuα* (a) or *vhuβ* (b) and purified Fur in the presence of $MnCl_2$ (top) or 1 mM EDTA as a chelator (bottom). (a) Lanes 1 to 5 represent Fur concentrations of 0, 150, 300, 500, and 700 nM, respectively. Lanes 6 to 8 represent 700 nM Fur with non-labeled probes as a competitor at 10, 50, and 100 ng, respectively. (b) Lanes 1 to 5 represent Fur concentrations of 0, 50, 100, 200, and 400 nM, respectively. Lanes 6 to 8 represent 400 nM Fur with non-labeled probes as a competitor at 10, 100, and 150 ng, respectively.

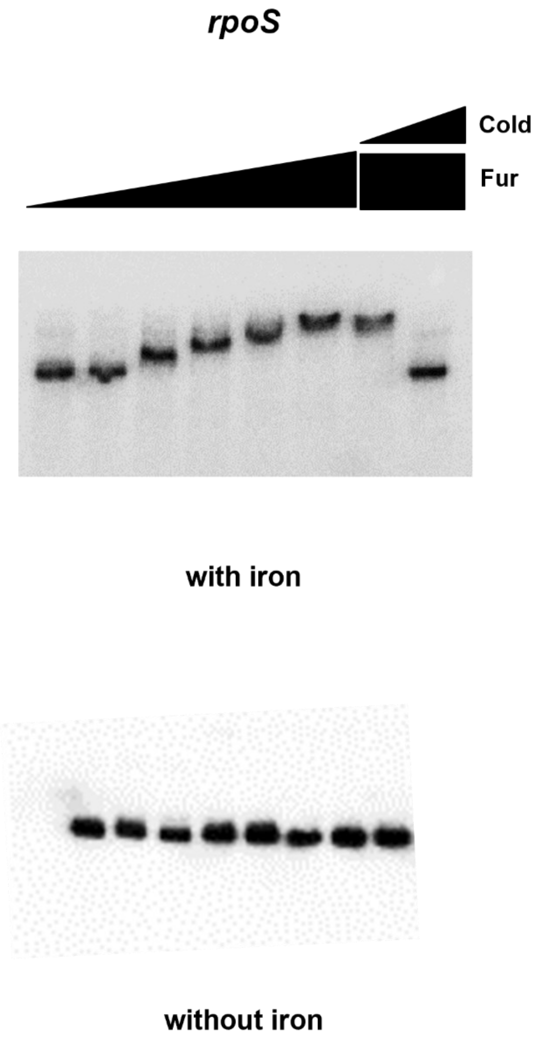

Supplementary Figure S4. Fur binding to the upstream region of *rpoS*.

Gel shift assays were performed using the radiolabeled 650-bp fragment from the upstream region of *rpoS* and purified Fur in the presence of $MnCl_2$ (top) or 1 mM EDTA as a chelator (bottom). Lanes 1 to 6 represent Fur concentrations of 0, 50, 100, 200, 300, and 500 nM, respectively. Lanes 7 and 8 represent 500 nM Fur with unlabeled probes as a competitor at 10 and 100 ng, respectively.

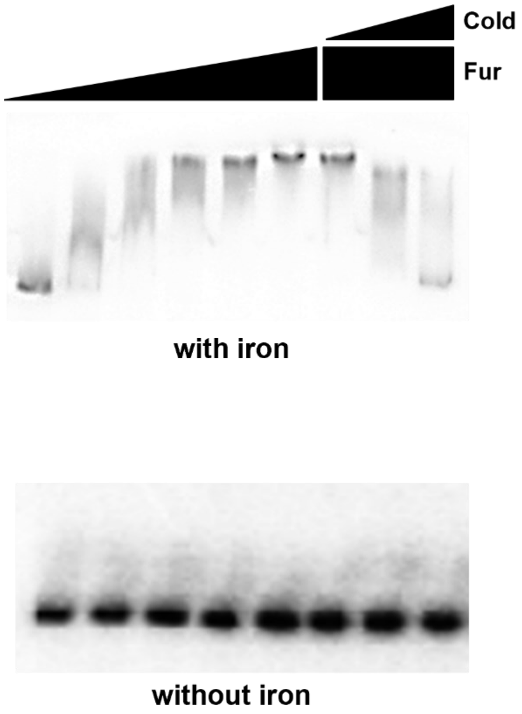

Supplementary Figure S5. Fur binding to the upstream region of *katG*.

Gel shift assays were performed using the radiolabeled 495-bp fragment from the upstream region of *katG* and purified Fur in the presence of $MnCl_2$ (top) or 1 mM EDTA as a chelator (bottom). Lanes 1 to 7 represent Fur concentrations of 0, 100, 200, 300, 400, 500, and 600 nM, respectively. Lanes 8 to 10 represent 600 nM Fur with unlabeled probes as a competitor at 10, 50, and 100 ng, respectively (up). Lanes 1 to 5 represent Fur concentrations of 0, 100, 200, 300, and 500 nM, respectively. Lanes 6 to 8 represent 500 nM Fur with unlabeled probes as a competitor at 10, 50, and 100 ng, respectively (down).

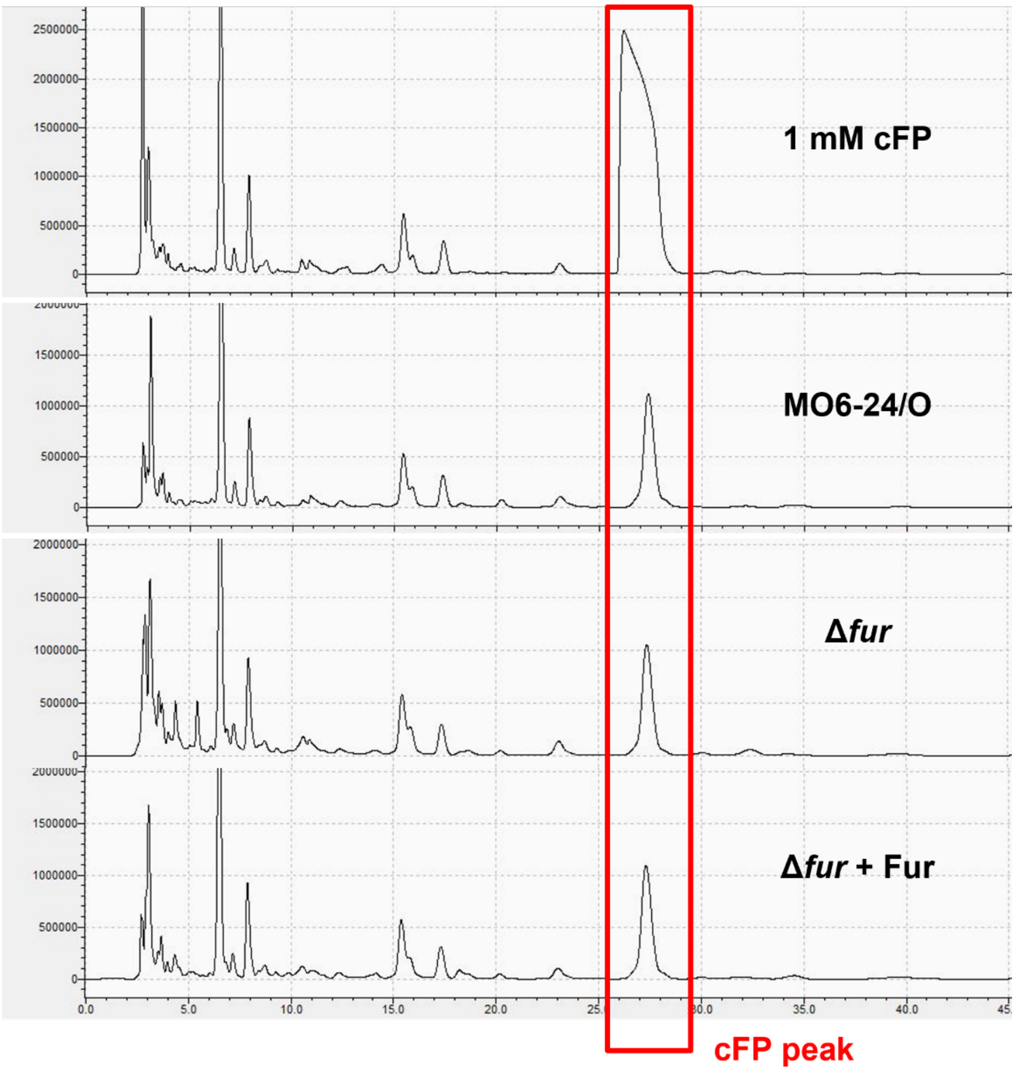

Supplementary Figure S6. The production of cFP in *V. vulnificus* is not influenced by Fur.

HPLC profiles were generated to analyze cFP production in three groups: MO6-24/O, Δ*fur*, and Δ*fur*+pRK-*fur*. As a control, 1 mM cFP was also included. The amount of cFP produced by each strain was estimated using the method as described in the Materials and Methods section.

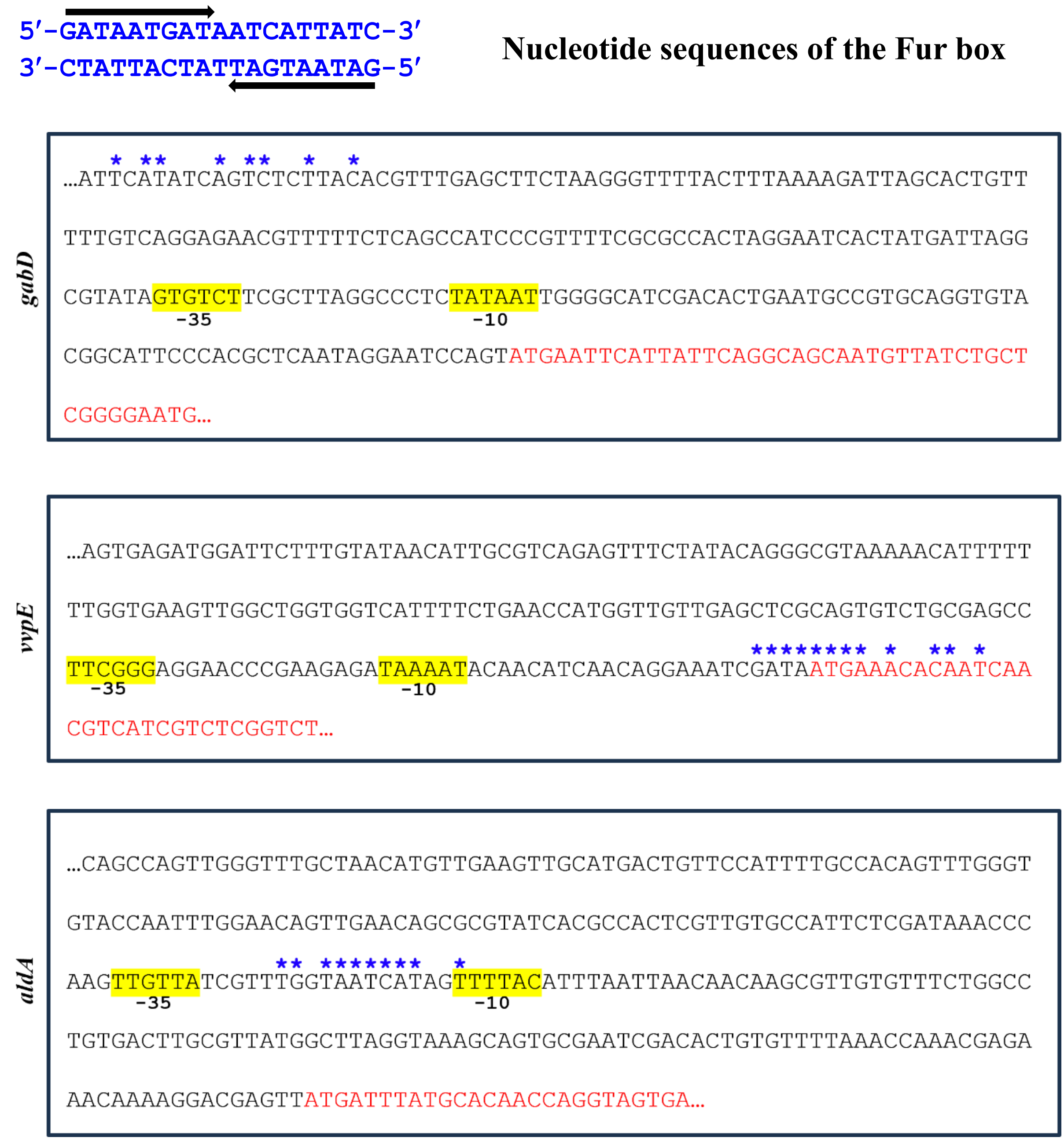

Supplementary Figure S7. The nucleotide sequences of the upstream regions of *rpoS*-related genes.

Nucleotide sequences of the upstream regions of three genes: *gabD* (top), *vvpE* (middle), and *aldA* (bottom). The promoter sequences (-35 and -10 regions) are highlighted, and the coding regions are indicated in red letters. Some sequences partly homologous to the Fur box are marked with asterisks. However, these sequences lack in the typical palindromic sequences of the Fur box.

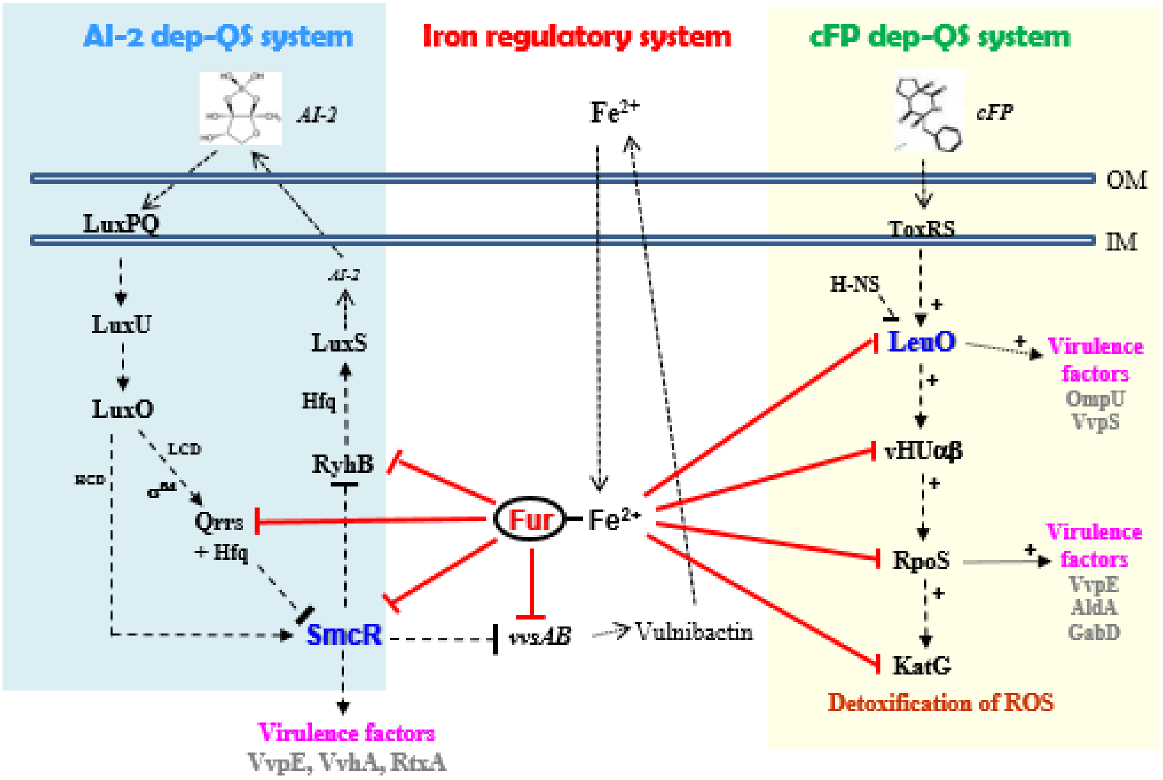

Supplementary Figure S8. Schematic overview of Fur-iron-mediated control in quorum-sensing regulatory pathways in *V. vulnificus*.

The *vvsAB* genes, responsible for encoding the iron siderophore vulnibactin, are repressed by the Fur-iron complex (Wen et al., J. Biol. Chem. 2012, 287: 26727). Additionally, the AI-2 QS system is downregulated by Fur-iron through repression of the small RNAs Qrrs (Wen et al., J. Biol. Chem. 2016, 291: 14213) and the master regulator SmcR (Kim et al., Infect. Immun. 2013, 81:2888). Fur-iron also exerts the repression on the small RNA RyhB, leading to the inhibition of LuxS, which encodes AI-2 biosynthesis (Lee et al. Sci. Rep. 2022, 312: 831). This study has demonstrated that Fur-iron affects components of the cFP-dependent QS system, thereby repressing downstream virulence factors. Abbreviations: HCD, high cell density; LCD, low cell density; OM, outer membrane; IM, inner membrane.
